# Cancer-Related Psychological Distress in Lymphoma Survivor: An Italian Cross-Sectional Study

**DOI:** 10.3389/fpsyg.2022.872329

**Published:** 2022-04-26

**Authors:** Giulia Agostinelli, Barbara Muzzatti, Samantha Serpentini, Michele Spina, Maria Antonietta Annunziata

**Affiliations:** ^1^Unit of Oncological Psychology - Centro di Riferimento Oncologico di Aviano (CRO), IRCCS, Aviano, Italy; ^2^IOV - Istituto Oncologico Veneto - IRCCS, Padua, Italy; ^3^Unit of Oncological Medicine - Centro di Riferimento Oncologico di Aviano (CRO), IRCCS, Aviano, Italy

**Keywords:** psychological distress, anxiety, depression, quality of life, lymphoma, survivor

## Abstract

Cancer is becoming a chronic disease, and the number of cancer survivors continues to increase. Lymphoma survivors are also increasing in numbers, and anxiety and depression are among the consequences they face. This study aimed to explore psychological distress in a sample of 212 lymphoma survivors. Information through a socio-demographic form and the compilation of questionnaires to assess anxiety, depression, quality of life, and the impact of cancer on lymphoma survivors was collected and analyzed. In the sample examined, 17% of lymphoma survivors were anxiety caseness, and 12.3% were depression caseness, and of these, 8% presented with concomitant anxiety depression. This study identified some variables associated with psychological distress in lymphoma survivors: female sex; living as a couple; a diagnosis of Hodgkin lymphoma; systematic treatment and/or radiotherapy; sleep disorders; no regular physical activity; and present or past use of psychiatric drugs. Our cross-sectional study results suggest that some of the variables investigated may be useful in identifying lymphoma survivors who are more likely to report psychological distress. It is important to monitor psychological distress along the entire trajectory of survivorship in order to identify early the presence of anxiety and depression and to provide timely psychological support.

## Introduction

The survival rate of cancers continues to rise as cancer increasingly turns into a chronic disease. It is estimated that, in 2018, 43.8 million cancer survivors, diagnosed in the previous 5 years, were alive worldwide (The American Cancer Society, Inc, 2019). In Italy, specifically, the number of people living after a diagnosis of cancer increases by about 3% annually (Guzzinati et al., 2018).

Advances in modern oncology and increased patient survival rates have triggered, in healthcare professionals and researchers, a necessary shift of attention toward survival management. In fact, survival is a dynamic dimension that moves along a continuum, and several studies indicate that having experienced an oncological disease leads to medium- and long-term consequences that can translate into physical, psychological, and social problems. The survival rate has also improved in patients with lymphoma, but the side effects and consequences that this type of survivors face are considerable. Studies show that patients with lymphoma have a greater intensity of psychological distress in the treatment phase than lymphoma survivors (Smith et al., 2009; Troy et al., 2019). However, it was also found that anxiety and depression are among the consequences experienced by cancer survivors and that their levels change with the passage of time (Raphael et al., 2019; Troy et al., 2019). Therefore, it is important to assess psychological distress along the entire survival trajectory and offer timely responses of adequate psychological support. In this regard, the guidelines emphasize the importance of carrying out an adequate assessment of the psychological distress at each checkup (Denlinger et al., 2016), but unfortunately, this activity is not yet widespread in clinical practice.

Many cancer survivors do not meet the criteria for significant psychological morbidity but still experience psychological distress (PDQ Supportive and Palliative Care Editorial Board, 2021). Distress has been defined as “a multifactorial unpleasant experience of a psychological (i.e., cognitive, behavioral, emotional), social, spiritual, and/or physical nature that may interfere with the ability to cope effectively with cancer, its physical symptoms, and its treatment” (Riba et al., 2019, p. 2).

Psychological distress is usually detected by measuring both anxiety and depression, and a high value of anxiety and/or depression is defined as high psychological distress (Loge et al., 1997; Aksnes et al., 2007; ACTION Study Group, 2017; Arts et al., 2018, 2021). Lack of detection of anxiety and depression has been shown to significantly affect the quality of life (QoL) and even increase mortality (Cuijpers and Smit, 2002; Pinquart and Duberstein, 2010; Mols et al., 2013).

However, there is a lack of agreement on the definition of survivorship (Annunziata and Muzzatti, 2014). The topic has been less studied, and the literature shows that studies analyzing psychological distress in lymphoma survivors who have finished treatments for at least 5 years are scarce and frequently show non-homogeneous results and do not reach statistical significance (Gil-Fernández et al., 2003; Aksnes et al., 2007; Bøhn et al., 2019). Furthermore, data must be extrapolated from survivor samples that include different types of cancer (Linden et al., 2012; ACTION Study Group, 2017; Bevilacqua et al., 2018; Götze et al., 2020; Joshy et al., 2020) or in hematological cancer generic samples (Korszun et al., 2014; Oberoi et al., 2017; Kuba et al., 2019; Raphael et al., 2019). For example, Kuba et al. (2019) found higher depression and anxiety symptoms in hematological cancer long-survivors than in the comparison group, whereas Oberoi et al. (2017) found that the levels of anxiety and depression turn out to be different when compared with the different types of hematological cancer.

In general, the literature indicates a higher prevalence of anxiety and depression and poor QoL in lymphoma survivors compared with other cancer survivors (ACTION Study Group, 2017; Joshy et al., 2020) and with other normative populations (Pettengell et al., 2008; Bellizzi et al., 2009; Oerlemans et al., 2014) with the prevalence rates of depression between 2 and 35% (Hjermstad et al., 2004; Wang et al., 2013) and of anxiety between 12 and 42% (Hjermstad et al., 2004; Thompson et al., 2010). However, to date, only few studies have been carried out, and therefore, the availability of evidence on the subject is scarce. Therefore, this study had the purpose to analyze a sample of lymphoma survivors who had finished treatments for at least 5 years, adopting the accepted Italian definition of survivorship (Annunziata and Muzzatti, 2014). In particular, the study aimed to describe their psychological distress in terms of anxiety and depression.

## Materials and Methods

### Setting and Population

A cross-sectional study was conducted on a sample of lymphoma survivors enrolled from 2017 to 2019 during the routine follow-up visit.

The patient inclusion criteria were 18 years of age at the time of diagnosis; free from disease and from having finished the treatments for at least 5 years; language skills (written and spoken) to be able to understand and complete the questionnaires; and signing of the consent form.

The patient exclusion criteria were history of psychiatric illness and/or evidence of psychiatric pathology; the presence of physical and/or sensory disabilities interfering with the accessibility of materials; and non-adherence to the inclusion criteria. The study was conducted according to the guidelines of the Declaration of Helsinki and was approved by the relevant ethics committee.

### Study Measures

After having presented the aims and objectives of the project and having received written consent to participate, a psychologist collected information through a socio-demographic form and the compilation of several questionnaires to assess anxiety, depression, QoL, and the impact of cancer on lymphoma survivors.

The socio-demographic form collected information with regard to age, marital status, education level, employment status, cancer-related variables, lifestyle, and two questions about psychological support and psychiatric drug. This information was collected through closed-ended questions, and the corresponding categories are shown in Tables 1–4.

**TABLE 1:** Socio-demographic variables.

Socio-demographic variables	Total sample, *n* = 212
Gender, *n* (%)	
Male	117 (55.2)
Female	95 (44.2)
Age in years at survey	
Mean (SD)	56.83 (13.55)
Median (min–max.)	54 (28–89)
Age at survey divided into classes, *n* (%)	
23–35	9 (4.3)
36–55	99 (46.7)
56–75	85 (40.1)
>75	19 (8.9)
Marital status divided in single/couple, *n* (%)	
Living as a couple	154 (72.6)
Single	58 (27.4)
Education level, *n* (%)^†^	
5 years	31 (14.6)
8 years	62 (29.3)
13 years	80 (37.7)
>13 years	39 (18.4)
Employment status, *n* (%)	
Active	117 (55.2)
Inactive	95 (44.8)

*^†^Study years.*

The questionnaires examined for this discussion are described below:

– Hospital Anxiety and Depression Scale (HADS, Zigmond and Snaith, 1983) is a tool frequently used for detecting psychological distress in patients with cancer (Muzzatti and Annunziata, 2012; Annunziata et al., 2020). HADS is a questionnaire validated in Italy (Costantini et al., 1999), which measures the levels of symptoms of anxiety and depression in two subscales of seven items, each of which showed good acceptability and reliability (Herrmann, 1997). In a recent article, it was reported that the HADS can be used to accurately determine the prevalence of anxiety and depression in patients with cancer (Annunziata et al., 2020). The cutoff used is >9 units to identify anxiety caseness (HADS-A) and >7 units for depression caseness (HADS-D) (Annunziata et al., 2020).
– The Impact of Cancer (IOC) Scale (Zebrack et al., 2006) is a self-assessment tool designed to measure the specific and multidimensional aspects of long-term cancer survival. It includes 81 items divided into different thematic areas, and the Italian version outlines three factors (Muzzatti et al., 2013), namely, I-Uncertainty/worry about health and future; II-Personal growth and altruism; and III-Dissatisfaction and life interferences.
– The 36-Item Short Form Health Survey Questionnaire (SF-36) (Ware et al., 1993) is a generic measure of QoL widely used in the general population validated in Italian (Apolone et al., 1997). It consists of 36 items that collect information on eight different QoL indices, namely, Physical Functioning; Role-Physical Limitation; Bodily Pain; General Health; Vitality; Social Functioning; Role-Emotional Limitation; and Mental Health.

### Data Collection

The collected protocols data were anonymized by assigning a progressive number to each subject and were stored in a dedicated database.

### Analysis

Anxiety and depression intensities were calculated, respectively, by totaling the HADS-A and HADS-D items. Differences in intensity between subgroups of participants (described below) were tested for significance with either Student’s *t*-test or a one-way univariate ANOVA followed by Bonferroni’s *post-hoc* test. Anxiety and depression intensities were compared using a paired Student’s *t*-test. The prevalence of both anxiety and depression in the sample was obtained using the case-finding criteria for HADS provided by Annunziata et al. (2020). Possible correlations between anxiety (or depression) scores and both the impact of cancer (assessed by IOC) and QoL (assessed by SF-36) were tested using Pearson’s correlation coefficients. In all analyses, *p* < 0.05 (two-tailed) was preset for statistical significance. Statistical Package for the Social Sciences (SPSS.21) was used to perform the analyses. There were no missing data.

### Institutional Review Board Statement

The study was conducted according to the guidelines of the Declaration of Helsinki and was approved by the Ethics Committee of Centro di Riferimento Oncologico di Aviano (CRO) IRCCS (protocol code: 21456–date of approval: September 16, 2016). Informed consent was obtained from all subjects involved in the study. Written informed consent has been obtained from the patients to publish this study.

## Results

### Study Sample

The study sample included 212 lymphoma survivors (117 male patients and 95 female patients). There were no refusals. The mean age at the time of the survey was 56.8 (SD 13.5) years. Age at survey was also divided into classes. Socio-demographic characteristics of the sample are summarized in Table 1.

Regarding cancer-related variables, 102 survivors were diagnosed with Hodgkin lymphoma (HL) and 110 survivors were diagnosed with non-Hodgkin lymphoma (NHL). The mean age at the time of diagnosis was 44.11 (SD 14.9) years, and the average time elapsed from diagnosis was 11 (SD 1.4) years. Since the survivors were enrolled at different times of follow-up, we divided them into classes based on the time since diagnosis. The treatments performed were divided into 7 categories. Most of the patients appeared to have undergone systematic treatment and/or radiotherapy (*n* = 141), while 46 patients underwent systematic treatment only, and 12 patients underwent surgery and systematic treatment and/or radiotherapy. The other categories involved fewer patients. Details of all cancer-related variables investigated are summarized in Table 2.

**TABLE 2:** Cancer-related variables.

Cancer-related variables	Total sample, *n* = 212
Type of lymphoma, *n* (%)	
HL	102 (48.1)
NHL	110 (51.9)
Age in years at diagnosis	
Mean (SD)	44.11 (19.94)
Median (min–max)	41.5 (18–81)
Age at diagnosis divided into classes, *n* (%)	
18–30	46 (21.7)
31–50	93 (43.9)
51–70	65 (30.6)
>70	8 (3.8)
Time since diagnosis divided into classes, *n* (%)	
5–9	80 (37.7)
10–14	65 (30.6)
15–19	30 (14.2)
≥20	37 (17.5)
Time since diagnosis (years)	
Mean (SD)	11 (1.41)
Median (min–max)	11 (5–37)
Treatment modality, *n* (%)	
Limited surgery	5 (2.4)
Systematic treatment alone	46 (21.7)
Systematic treatment and/or Radiotherapy	141 (66.5)
Surgery and systematic treatment and/or Radiotherapy	12 (5.6)
Transplant and systematic treatment and/or Radiotherapy	4 (1.9)
Hormone therapy and systematic treatment and/or Radiotherapy	3 (1.4)
No treatment	1 (0.5)

Some areas related to the survivors’ lifestyle were then investigated: eating style, sleep, and physical activity; 18 survivors reported irregular/problematic eating, and 81 survivors reported sleep-related problems; 97 survivors reported regular physical activity, while 51 survivors reported no physical activity at all. Data are summarized in Table 3.

**TABLE 3:** Lifestyle variables.

Lifestyle variables	Total sample, *n* = 212
Eating style, *n* (%)	
Regular^†^	194 (91.5)
Irregular and/or problematic^‡^	18 (8.5)
Sleep, *n* (%)	
Regular sleep	131 (61.8)
Difficulty falling asleep/waking up	22 (10.4)
Disturbed sleep	47 (22.2)
Unrefreshing sleep	12 (5.6)
Physical activity, *n* (%)	
Regular^§^	97 (45.7)
Irregular^¶^	64 (30.2)
Absent	51 (24.1)

*^†^Regular meal scanning.*

*^‡^Irregular meal scanning and/or objective difficulties in feeding (allergies and intolerances).*

*^§^At least one time a week.*

*^¶^Occasional.*

In the two questions about having received some form of psychological support and having used psychiatric drugs without having received a diagnosis of psychiatric disorder (now or in the past), the patients answered for the majority that they had received some form of psychological support and had not used psychiatric drugs. Data are shown in Table 4.

**TABLE 4:** Psychological support and psychiatric drugs.

Psychological support and psychiatric drugs	Total sample, *n* = 212
Psychological support (now or in the past)^†^, *n* (%)	
Yes	130 (61.3)
No	82 (38.7)
Use of psychiatric drugs (now or in the past)^‡^, *n* (%)	
Yes	43 (20.3)
No	169 (79.7)

*^†^Questions about having received some form of psychological support without having received a diagnosis of psychiatric disorder.*

*^‡^Questions about having used psychiatric drugs without having received a diagnosis of psychiatric disorder.*

### Prevalence of Anxiety and/or Depression in Lymphoma Survivors and Anxiety and Depression Intensity

In the sample examined, 17% of lymphoma survivors were anxiety caseness and 12.3% were depression caseness. Cross-tabulation of anxiety caseness and depression caseness found that 9% presented exclusively anxiety, 4.2% presented exclusively depression, and 8% presented concomitance of anxiety and depression. In the sample examined, lymphoma survivors showed higher anxiety intensity than the reported depression intensity; in particular, the average anxiety and depression intensities were 5.4 (SD: 3.7) and 4.0 (SD: 3) (*p* < 0.001), respectively.

### Psychological Distress and Its Association With the Different Variables Analyzed

After assessing the cohort prevalence of anxiety and depression, socio-demographic characteristics, cancer-related variables, lifestyle, and psychological support/psychiatric drugs use were assessed in the percentage of the cohort having anxiety and depression caseness. All the variables are described below.

#### Psychological Distress and Its Association With Demographic Variables

With respect to gender variable, the analyses showed that women had higher values than men both in anxiety (M: 6.8, SD: 3.8 vs. M: 4.8, SD: 3.4; *p* < 0.001) and in depression intensities (M: 4.5, SD: 3.1 vs. M: 3.6, SD: 2.8; *p* = 0.027). Analyzing age at survey divided into classes, no significant differences emerged for anxiety (*p* = 0.149) and depression (*p* = 0.613) intensities in the different age groups. For marital status, significant data emerged: individuals living as a couple had higher intensities of both anxiety (M: 6.1, SD: 4.0; *p* = 0.009) and depression (M: 4.3, SD: 3.1; *p* = 0.010) than the intensities of anxiety (M: 4.6, SD: 2.4) and depression (M: 3.1, SD: 2.3) of those who were single. On the contrary, no significant differences emerged with respect to anxiety and depression compared with the education level and employment status (*p* > 0.05).

#### Psychological Distress and Its Association With Cancer-Related Variables

Analyzing age at diagnosis divided into classes, no significant differences emerged in the intensities of anxiety (*p* = 0.178) and depression (*p* = 0.387) in the different age groups. Similarly, no significant differences were found in the time since diagnosis was divided into classes (*p* > 0.05) in the different survivorship groups. However, it appeared that, as survivorship progressed, anxiety intensity increased (*R* = 0.711, *p* < 0.001) but not depression intensity (*R* = 0.128, *p* = 0.063). With respect to the types of lymphoma, a significant difference emerged only for anxiety, which was greater in HL (M: 6.3, SD: 3.6) compared with NHL (M: 5.2, SD: 3.8) (*p* = 0.037). Regarding the type of treatment, both anxiety (M: 6.3, SD: 3.8; *p* = 0.002) and depression (M: 4.3, SD: 3.2; *p* = 0.019) were significantly higher only in lymphoma survivors who had undergone systematic treatment and/or radiotherapy compared with other types of treatment.

#### Psychological Distress and Its Association With Lifestyle

For the variables related to lifestyle, no significant differences emerged in anxiety (*p* = 0.216) and depression (*p* = 0.424) intensities in the eating style. Analyzing the sleep variable, significant results emerged for both anxiety (*p* = 0.006) and depression (*p* < 0.001). In particular, subjects who reported having unrefreshing sleep showed a higher intensity of anxiety than subjects who reported having regular sleep (M: 8.4, SD: 4.2 vs. M: 5.1, SD: 3.1) (*p* = 0.001). Instead, for the depression, “regular sleep” survivors showed a lower intensity of depression (M: 3.3, SD: 2.5) than survivors in all other sleep categories, such as “difficulty falling asleep/waking up” (M: 5.6, SD: 3.3; *p* < 0.001), “disturbed sleep” (M. 4.8, SD: 3.6; *p* = 0.003), and “unrefreshing sleep” (M: 5.6, SD: 2.4; *p* = 0.004).

Concerning the physical activity variable, differences emerged in the intensities of both anxiety (*p* = 0.005) and depression (*p* = 0.007) based on the frequency of physical activity performed. In particular, subjects who did not perform physical activity reported a higher intensity of anxiety (M: 7.2, SD: 3.9) than survivors who regularly engaged in physical activity (M: 5.2; SD: 3.3) (*p* = 0.001). Similarly for depression, the differences reached statistical significance (*p* = 0.003) by comparing the subgroups of those who did not perform physical activity (M: 5.0, SD: 3.7) with those who performed a regular physical activity (M: 3.4, SD: 2.5).

#### Psychological Distress and Its Association With Psychological Support and Use of Psychoactive Drugs

No significant differences emerged in the anxiety (*p* = 0.855) and depression (*p* = 0.609) intensities in those who received some form of psychological support compared with those who did not receive it. Instead, there was a significant difference for both anxiety (M 8.3, SD: 4.3 vs. M: 5.1, SD: 3.2; *p* < 0.001) and depression (M: 6.0, SD: 3,2 vs. M: 3.5, SD: 2.7; *p* < 0.001) scores between those who used psychiatric drugs in the past vs. who had never used it.

### Association of Anxiety and Depression With Quality of Life and Impact of Cancer

Finally, the correlations of anxiety and depression with the IOC (Table 5) and the QoL (Table 6) were investigated. Anxiety and depression were positively correlated with factor I (uncertainty/worry about health and future) and with factor III (dissatisfaction and life interferences) of the IOC; no type of correlation with factor II (personal growth and altruism) emerged instead. Anxiety and depression scores were significantly negatively correlated with all dimensions of QoL investigated by SF-36.

**TABLE 5:** Correlations of anxiety and depression with IOC.

IOC	Anxiety	Anxiety	Depression	Depression
	R	P	R	P
Factor I IOC^†^	0.565	0.01	0.406	0.01
Factor II IOC^‡^	0.110	–	0.005	–
Factor III IOC^§^	0.461	0.01	0.391	0.01

*^†^Uncertainty/worry about health and future.*

*^‡^Dissatisfaction and life interference.*

*^§^Personal growth and altruism.*

**TABLE 6:** Correlations of anxiety and depression with QoL.

SF-36	Anxiety	Anxiety	Depression	Depression
	R	P	R	P
Physical functioning	− 0.354	0.01	− 0.307	0.01
Role-physical limitation	− 0.289	0.01	− 0.310	0.01
Bodily pain	− 0.450	0.01	− 0.348	0.01
General health	− 0.508	0.01	− 0.431	0.01
Vitality	− 0.565	0.01	− 0.605	0.01
Social functioning	− 0.635	0.01	− 0.633	0.01
Role-emotional limitation	− 0.528	0.01	− 0.546	0.01
Mental health	− 0.662	0.01	− 0.614	0.01

## Discussion

The need to carry out this study took inspiration from the observation that in the literature, few studies have analyzed psychological distress in lymphoma survivors who were at least 5 years after the end of treatments, and frequently, data must be extrapolated from generic cancer and/or hematological survivor samples (Linden et al., 2012; Korszun et al., 2014; ACTION Study Group, 2017; Oberoi et al., 2017; Bevilacqua et al., 2018; Kuba et al., 2019; Raphael et al., 2019; Götze et al., 2020; Joshy et al., 2020). It is also important to consider that studies often speak generically of psychological distress without distinguishing the components of anxiety and depression (Korszun et al., 2014; Jones et al., 2015; Raphael et al., 2019; Troy et al., 2019; Joshy et al., 2020). In addition, the literature shows that lymphoma survivors often have specific characteristics (such as: <QoL, pain, depression, and anxiety) because the clinical processes and therapies they undergo produce strong adverse effects (Vargas-Román et al., 2020).

This cross-sectional study explores different variables of a sample of lymphoma survivors surveyed from 2017 to 2019, who were at least 5 years from the end of the treatments. The aim of this study was to identify variables associated with psychological distress. Some results confirmed what was expected, and others moved in the opposite direction from what had been hypothesized and from the literature.

The incidence of anxious and depressive symptoms in the lymphoma survivor does not always appear homogeneous, and often, they are not statistically significant (Gil-Fernández et al., 2003; Aksnes et al., 2007; Bøhn et al., 2019). In our study, the prevalence of anxiety (17%) and depression (12.3%) found in the total sample of lymphoma survivors is, however, in line with the data found in the literature (Hjermstad et al., 2004; Thompson et al., 2010; Wang et al., 2013), underlining how the survivorship condition of this specific illness is characterized by consequences that increase the levels of psychological distress (Korszun et al., 2014) compared with other types of tumors (Joshy et al., 2020). In accordance with what was reported in the literature, our sample of lymphoma survivors showed higher anxiety intensity than the depression intensity (Loge et al., 1997; Gil-Fernández et al., 2003; Aksnes et al., 2007; Daniëls et al., 2014; Ng et al., 2016; Husson et al., 2017; Magyari et al., 2017; Bøhn et al., 2019).

By comparing the anxiety and depression intensities in male and female lymphoma survivors, in line with what was reported in the literature, we found higher values of both anxiety (Loge et al., 1997; Gil-Fernández et al., 2003; Götze et al., 2020) and depression in women (Götze et al., 2020).

Contrary to what was expected and to what literature indicates, namely, that single survivors have a higher intensity of distress (Loge et al., 1997; Jensen et al., 2013; Jones et al., 2015), we have found higher rates of anxiety and depression in those who lived as a couple than in those who were single. This result made us hypothesize that caregivers could be, in some circumstances, an additional source of stress and concern for the patient and therefore should be adequately supported (Sklenarova et al., 2015; Kemp et al., 2018).

Conflicting data also emerged with respect to the literature regarding the association between higher psychological distress intensity and inactivity in employment or a lower education level (Loge et al., 1997; Aksnes et al., 2007; Ng et al., 2016; Magyari et al., 2017). Indeed, our data showed no significant differences in the intensity of psychological distress by comparing the employment status and the education level.

All the variables relating to age (age at diagnosis divided into classes and age at survey divided into classes) and timing (time since diagnosis divided into classes) did not report significant differences in anxiety and depression. Moreover, different data are found in the literature but the comparison has limited validity as the studies are not specific to lymphoma survivors. In some studies, it was observed that younger survivors experience higher intensities of anxiety and depression than older survivors (Jensen et al., 2013; Jones et al., 2015; Kuba et al., 2019; Götze et al., 2020); other studies show that young survivors have a higher intensity of anxiety and the elderly have higher intensity of depression (Loge et al., 1997; Gil-Fernández et al., 2003; Oerlemans et al., 2014; Ng et al., 2016). The comparison between the age at diagnosis and the anxiety and depression intensities is less studied in the literature, and only data related to the age at the time of the survey and the years elapsed since diagnosis are found. With respect to the timing of survivorship, some studies found no significant differences between long-term and short-term survivorship (Smith et al., 2009; Troy et al., 2019; Götze et al., 2020). Some show that both anxiety and depression intensities increase over time (Loge et al., 1997; Oerlemans et al., 2014); other studies show that the intensity of depression increases while that of anxiety decreases (Kuba et al., 2019), highlighting how depression, more than anxiety, represents a problem of long-term survivorship.

It is interesting to note that, by comparing the two different types of diagnoses (HL vs. NHL), higher values of both anxiety and depression emerged in the subgroup of HL survivors, differences that reached statistical significance only for anxiety. Regarding these data, useful references were not found in the literature where lymphomas are generally analyzed either separately or in a single sample but not in comparison. However, the data found made us ponder on the different impacts that these diseases can have on survivors and reflect on the usefulness of future studies that consider this difference.

For the type of treatment received, it was observed that those who had performed systematic treatment and/or radiotherapy had higher intensities of anxiety and depression than those who had received other treatment types. To this regard, Arts et al. (2018) also have identified how survivors who underwent systemic treatment received a greater number of psychosocial interventions. However, in studies investigating psychological distress in lymphoma survivors, the type of treatment performed is often not reported, or data are not significant (Loge et al., 1997; Gil-Fernández et al., 2003). Most of the studies that take into consideration the type of treatment have primarily focused on autologous transplantation finding; in some cases, higher depression was observed in those who had undergone autologous transplantation (Gil-Fernández et al., 2003; Rusiewicz et al., 2008), and in other cases, no difference was observed from the general population 1 year after the autologous transplantation (Hjermstad et al., 1999, 2004).

Compared to the information collected on the lifestyle, no significant differences emerged in the intensity of psychological distress in relation to eating style, and we have not found specific studies that investigate the eating style in lymphoma survivors.

Instead, significant differences emerged in the anxiety and depression intensities in relation to the sleep variable: those who had a regular sleep pattern presented a lower intensity of anxiety than those who experienced an unrefreshing sleep, and those who had a regular sleep pattern presented lower intensity of depression than those who had disturbed sleep, unrefreshing sleep, and difficulty falling asleep/waking up. A recent study (Slade et al., 2020) found that 34% of cancer survivors had sleep disturbance, compared with 23% of non-cancer patients in the general population with no history of cancer (*p* < 0.001). However, in the literature, there is less evidence relating to the variable of sleep in lymphoma survivors and slightly more evidence for generic cancer survivors, underlining how this is less studied and taken into consideration. The problems most reported by cancer survivors seem to be problems of falling asleep (Andrykowski et al., 1998), difficulties of sleep maintenance (Engstrom et al., 1999), and sleep duration (Savard and Morin, 2001). Also, sleep disorders are usually studied mainly in relation to depression alone (Irwin et al., 2013; Ng et al., 2016). In this regard, however, it must be specified that sleep-related difficulties are often reported as symptoms of both anxiety and depression, and such difficulties could therefore be manifestations of psychological distress rather than causes.

Finally, with respect to the variable of physical activity, significant differences emerged in the intensities of both anxiety and depression between individuals who regularly performed physical activity and those who did not. This is in line with the literature where it is stressed how physical activity can be a wellness tool to decrease psychological distress in patients with cancer and cancer survivors (Zhao et al., 2013). It is important to note that the absence of physical activity could also be a manifestation of patients’ mobility/ability problems (the SF-36 factors of physical role functioning, physical functioning, and bodily pain were all correlated with anxiety and depression), so it could be that patients with more depression and anxiety would be less likely to engage in physical activity because they are less able to perform it. Anyway, it is important to note that, even with regard to the dimension of physical activity, there is scarce literature, and it is studied more generally in cancer survivors and especially in the correlation with fatigue.

In our study, contrary to what was expected, the intensity of psychological distress reported by lymphoma survivors does not differ based on having received some form of psychological support. Compared with these data, only few studies investigate the psychological interventions in the population of lymphoma survivors. In general, it is highlighted that psycho-educational intervention and behavioral therapy appear to be effective in improving QoL, coping strategies, and self-efficacy and in reducing the symptoms of anxiety, depression, and psychological distress in cancer survivors (Park and Bae, 2017). Arch et al. (2018) have reported that cancer survivors rate individual professional counseling as their preferred form of psychological intervention and report significant unmet needs for psychological intervention. However, studies on this subject are scarce, and this aspect should be further investigated in the future.

In accordance with the literature (Loge et al., 1997), survivors who had used or made an occasional use of psychiatric drugs presented a higher intensity of psychological distress than those who did not use them. In this regard, Øvlisen et al. (2020) pointed out that patients diagnosed with HL were at a higher risk of receiving prescriptions for psychiatric drugs but that this increased risk was transient and normalized to the matched 5-year surviving population.

The correlations of anxiety and depression with the IOC and the QoL confirm what we expected and what is reported in the literature. Anxiety and depression were positively correlated with uncertainty, worry about health and future, dissatisfaction, and life interferences and were negatively correlated with all dimensions of QoL. In general, it was observed that, in cancer survivors, including lymphoma survivors, a higher intensity of psychological distress was correlated with a negative IOC score (Korszun et al., 2014; Sarker et al., 2017) and with lower QoL scores (Ng et al., 2016; Jefford et al., 2017).

In conclusion, the results of this study suggest that some of the variables investigated may be useful in identifying lymphoma survivors who are more likely to report a higher intensity of psychological distress. In particular, we found that the variables associated with a high intensity of psychological distress in lymphoma survivors are as follow: female sex, living as a couple, diagnosis of HL, having undergone systematic treatment and/or radiotherapy, having sleep disturbances, not performing regular physical activity, and having taken in the past or having occasionally taken psychiatric drugs.

These could be important indicators to consider in clinical work in monitoring psychological distress along the entire trajectory of survivorship in order to identify early the presence of anxiety and depression and provide timely psychological support. Being an exploratory study, we can describe the relationships that emerged but cannot explain the differences, and this is definitely a limitation, along with the fact that it is a cross-sectional study, and therefore, the study acquired data only at a single point in time. It is also important to note that, after cancer treatment ends, there may be other life events that contribute to anxiety and depression and that the results are based on a small percentage of the cohort having anxiety caseness (17%) and depression caseness (12%). Strengths of the study include a good number in the study cohort and no refusals. In addition, demographic data appeared well matched between gender, age, and lymphoma type, and this adds strength to the findings. The results raise some interesting points that have not been seen before in the literature and outline the need for further prospective and longitudinal studies. It emerges, in fact, the need to systematically investigate survivorship in consideration of the different timing of survivorship (short term and long term) and propose screening programs to monitor psychological distress throughout the duration of survivorship and to promptly address the needs experienced.

## Data Availability Statement

The original contributions presented in the study are included in the article/Supplementary Material, further inquiries can be directed to the corresponding author/s.

## Ethics Statement

This study was conducted according to the guidelines of the Declaration of Helsinki, and approved by the Ethics Committee of Centro di Riferimento Oncologico di Aviano (CRO) IRCCS (protocol code: 21456–date of approval: September 16, 2016). The patients/participants provided their written informed consent to participate in this study.

## Author Contributions

MA: conceptualization. MA and BM: methodology. BM: formal analysis. GA: investigation, data curation, and writing—original draft preparation. SS and BM: writing—review and editing. MA and MS: project administration and funding acquisition. All authors have read and agreed to the published version of the manuscript.

## Conflict of Interest

The authors declare that the research was conducted in the absence of any commercial or financial relationships that could be construed as a potential conflict of interest.

## Publisher’s Note

All claims expressed in this article are solely those of the authors and do not necessarily represent those of their affiliated organizations, or those of the publisher, the editors and the reviewers. Any product that may be evaluated in this article, or claim that may be made by its manufacturer, is not guaranteed or endorsed by the publisher.
