# Supplementary material for: Cancer-Related Psychological Distress in Lymphoma Survivor: An Italian Cross-Sectional Study
Source: Front Psychol. 2022 Apr 26;13:872329. doi: 10.3389/fpsyg.2022.872329 (PMC9088809; doi:10.3389/fpsyg.2022.872329)
Supplement: Supplementary file 1 [file Data_Sheet_1.zip › STATISTIC ANALYSIS/01_Frequencies_A_D.HTM]

<!--Text used as the document title (displayed in the title bar).-->


# Frequencies


Notes

| Output Created | | 26-DEC-2020 10:03:16 |
| Comments | |  |
| Input | Data | C:\Users\Barbara\cro\analisi\_dati\survivors\_linfomi\_dati2020\dati\_2020\_survivor\_linfoma\_n212.sav |
| Filter | <none> |
| Weight | <none> |
| Split File | <none> |
| N of Rows in Working Data File | 212 |
| Missing Value Handling | Definition of Missing | User-defined missing values are treated as missing. |
| Cases Used | Statistics are based on all cases with valid data. |
| Syntax | | FREQUENCIES  VARIABLES=caseness\_ansia caseness\_depressione  /ORDER= ANALYSIS . |
| Resources | Elapsed Time | 0:00:00,02 |
| Total Values Allowed | 149796 |

  


Statistics

|  |  | caseness\_ansia | caseness\_depressione |
| N | Valid | 212 | 212 |
| Missing | 0 | 0 |

  


# Frequency Table


caseness\_ansia

|  |  | Frequency | Percent | Valid Percent | Cumulative Percent |
| Valid | ,00 | 176 | 83,0 | 83,0 | 83,0 |
| 1,00 | 36 | 17,0 | 17,0 | 100,0 |
| Total | 212 | 100,0 | 100,0 |  |

  


caseness\_depressione

|  |  | Frequency | Percent | Valid Percent | Cumulative Percent |
| Valid | ,00 | 186 | 87,7 | 87,7 | 87,7 |
| 1,00 | 26 | 12,3 | 12,3 | 100,0 |
| Total | 212 | 100,0 | 100,0 |  |

  
