# Supplementary material for: Cancer-Related Psychological Distress in Lymphoma Survivor: An Italian Cross-Sectional Study
Source: Front Psychol. 2022 Apr 26;13:872329. doi: 10.3389/fpsyg.2022.872329 (PMC9088809; doi:10.3389/fpsyg.2022.872329)
Supplement: Supplementary file 1 [file Data_Sheet_1.zip › STATISTIC ANALYSIS/02_Descriptives_A_D.HTM]

<!--Text used as the document title (displayed in the title bar).-->


# Descriptives


Notes

| Output Created | | 26-DEC-2020 10:05:34 |
| Comments | |  |
| Input | Data | C:\Users\Barbara\cro\analisi\_dati\survivors\_linfomi\_dati2020\dati\_2020\_survivor\_linfoma\_n212.sav |
| Filter | <none> |
| Weight | <none> |
| Split File | <none> |
| N of Rows in Working Data File | 212 |
| Missing Value Handling | Definition of Missing | User defined missing values are treated as missing. |
| Cases Used | All non-missing data are used. |
| Syntax | | DESCRIPTIVES  VARIABLES=a\_hads\_a a\_hads\_d  /STATISTICS=MEAN STDDEV MIN MAX . |
| Resources | Elapsed Time | 0:00:00,02 |

  


Descriptive Statistics

|  | N | Minimum | Maximum | Mean | Std. Deviation |
| a\_hads\_a | 212 | 0 | 18 | 5,72 | 3,717 |
| a\_hads\_d | 212 | 0 | 16 | 4,01 | 2,983 |
| Valid N (listwise) | 212 |  |  |  |  |

  
