# Supplementary material for: Cancer-Related Psychological Distress in Lymphoma Survivor: An Italian Cross-Sectional Study
Source: Front Psychol. 2022 Apr 26;13:872329. doi: 10.3389/fpsyg.2022.872329 (PMC9088809; doi:10.3389/fpsyg.2022.872329)
Supplement: Supplementary file 1 [file Data_Sheet_1.zip › STATISTIC ANALYSIS/03_Crosstabs_A_D.HTM]

<!--Text used as the document title (displayed in the title bar).-->


# Crosstabs


Notes

| Output Created | | 26-DEC-2020 10:07:01 |
| Comments | |  |
| Input | Data | C:\Users\Barbara\cro\analisi\_dati\survivors\_linfomi\_dati2020\dati\_2020\_survivor\_linfoma\_n212.sav |
| Filter | <none> |
| Weight | <none> |
| Split File | <none> |
| N of Rows in Working Data File | 212 |
| Missing Value Handling | Definition of Missing | User-defined missing values are treated as missing. |
| Cases Used | Statistics for each table are based on all the cases with valid data in the specified range(s) for all variables in each table. |
| Syntax | | CROSSTABS  /TABLES=caseness\_ansia BY caseness\_depressione  /FORMAT= AVALUE TABLES  /CELLS= COUNT TOTAL  /COUNT ROUND CELL . |
| Resources | Elapsed Time | 0:00:00,03 |
| Dimensions Requested | 2 |
| Cells Available | 116508 |

  


Case Processing Summary

|  | Cases | | | | | |
| Valid | | Missing | | Total | |
| N | Percent | N | Percent | N | Percent |
| caseness\_ansia \* caseness\_depressione | 212 | 100,0% | 0 | ,0% | 212 | 100,0% |

  


caseness\_ansia \* caseness\_depressione Crosstabulation

|  |  |  | caseness\_depressione | | Total |
| ,00 | 1,00 |  

| caseness\_ansia | ,00 | Count | 167 | 9 | 176 |
| % of Total | 78,8% | 4,2% | 83,0% |
| 1,00 | Count | 19 | 17 | 36 |
| % of Total | 9,0% | 8,0% | 17,0% |
| Total | | Count | 186 | 26 | 212 |
| % of Total | 87,7% | 12,3% | 100,0% |

  
