# Supplementary material for: Cancer-Related Psychological Distress in Lymphoma Survivor: An Italian Cross-Sectional Study
Source: Front Psychol. 2022 Apr 26;13:872329. doi: 10.3389/fpsyg.2022.872329 (PMC9088809; doi:10.3389/fpsyg.2022.872329)
Supplement: Supplementary file 1 [file Data_Sheet_1.zip › STATISTIC ANALYSIS/04_Correlations_HADS_SF36_IOC.HTM]

<!--Text used as the document title (displayed in the title bar).-->


# Correlations


Notes

| Output Created | | 26-DEC-2020 10:11:41 |
| Comments | |  |
| Input | Data | C:\Users\Barbara\cro\analisi\_dati\survivors\_linfomi\_dati2020\dati\_2020\_survivor\_linfoma\_n212.sav |
| Filter | <none> |
| Weight | <none> |
| Split File | <none> |
| N of Rows in Working Data File | 212 |
| Missing Value Handling | Definition of Missing | User-defined missing values are treated as missing. |
| Cases Used | Statistics for each pair of variables are based on all the cases with valid data for that pair. |
| Syntax | | CORRELATIONS  /VARIABLES=a\_hads\_a a\_hads\_d SF36\_att\_fis SF36\_lim\_fis SF36\_dol\_fis SF36\_sal\_gen SF36\_vital SF36\_lim\_soc SF36\_lim\_emo  SF36\_sal\_men FATTORE1 FATTORE2 FATTORE3  /PRINT=TWOTAIL NOSIG  /MISSING=PAIRWISE . |
| Resources | Elapsed Time | 0:00:00,08 |

  


Correlations

|  |  | a\_hads\_a | a\_hads\_d | SF36\_att\_fis | SF36\_lim\_fis | SF36\_dol\_fis | SF36\_sal\_gen | SF36\_vital | SF36\_lim\_soc | SF36\_lim\_emo | SF36\_sal\_men | FATTORE1 | FATTORE 2 | FATTORE 3 |
| a\_hads\_a | Pearson Correlation | 1 | ,711(\*\*) | -,354(\*\*) | -,289(\*\*) | -,450(\*\*) | -,508(\*\*) | -,565(\*\*) | -,635(\*\*) | -,528(\*\*) | -,662(\*\*) | ,565(\*\*) | ,110 | ,461(\*\*) |
| Sig. (2-tailed) |  | ,000 | ,000 | ,000 | ,000 | ,000 | ,000 | ,000 | ,000 | ,000 | ,000 | ,111 | ,000 |
| N | 212 | 212 | 209 | 209 | 209 | 209 | 209 | 209 | 209 | 209 | 212 | 212 | 212 |
| a\_hads\_d | Pearson Correlation | ,711(\*\*) | 1 | -,307(\*\*) | -,310(\*\*) | -,348(\*\*) | -,431(\*\*) | -,605(\*\*) | -,633(\*\*) | -,546(\*\*) | -,614(\*\*) | ,406(\*\*) | ,005 | ,391(\*\*) |
| Sig. (2-tailed) | ,000 |  | ,000 | ,000 | ,000 | ,000 | ,000 | ,000 | ,000 | ,000 | ,000 | ,941 | ,000 |
| N | 212 | 212 | 209 | 209 | 209 | 209 | 209 | 209 | 209 | 209 | 212 | 212 | 212 |
| SF36\_att\_fis | Pearson Correlation | -,354(\*\*) | -,307(\*\*) | 1 | ,672(\*\*) | ,550(\*\*) | ,565(\*\*) | ,479(\*\*) | ,473(\*\*) | ,368(\*\*) | ,370(\*\*) | -,419(\*\*) | -,155(\*) | -,468(\*\*) |
| Sig. (2-tailed) | ,000 | ,000 |  | ,000 | ,000 | ,000 | ,000 | ,000 | ,000 | ,000 | ,000 | ,025 | ,000 |
| N | 209 | 209 | 209 | 209 | 209 | 209 | 209 | 209 | 209 | 209 | 209 | 209 | 209 |
| SF36\_lim\_fis | Pearson Correlation | -,289(\*\*) | -,310(\*\*) | ,672(\*\*) | 1 | ,545(\*\*) | ,519(\*\*) | ,467(\*\*) | ,474(\*\*) | ,458(\*\*) | ,327(\*\*) | -,401(\*\*) | -,122 | -,398(\*\*) |
| Sig. (2-tailed) | ,000 | ,000 | ,000 |  | ,000 | ,000 | ,000 | ,000 | ,000 | ,000 | ,000 | ,079 | ,000 |
| N | 209 | 209 | 209 | 209 | 209 | 209 | 209 | 209 | 209 | 209 | 209 | 209 | 209 |
| SF36\_dol\_fis | Pearson Correlation | -,450(\*\*) | -,348(\*\*) | ,550(\*\*) | ,545(\*\*) | 1 | ,412(\*\*) | ,473(\*\*) | ,498(\*\*) | ,411(\*\*) | ,340(\*\*) | -,379(\*\*) | -,095 | -,372(\*\*) |
| Sig. (2-tailed) | ,000 | ,000 | ,000 | ,000 |  | ,000 | ,000 | ,000 | ,000 | ,000 | ,000 | ,173 | ,000 |
| N | 209 | 209 | 209 | 209 | 209 | 209 | 209 | 209 | 209 | 209 | 209 | 209 | 209 |
| SF36\_sal\_gen | Pearson Correlation | -,508(\*\*) | -,431(\*\*) | ,565(\*\*) | ,519(\*\*) | ,412(\*\*) | 1 | ,566(\*\*) | ,584(\*\*) | ,412(\*\*) | ,464(\*\*) | -,595(\*\*) | -,076 | -,561(\*\*) |
| Sig. (2-tailed) | ,000 | ,000 | ,000 | ,000 | ,000 |  | ,000 | ,000 | ,000 | ,000 | ,000 | ,274 | ,000 |
| N | 209 | 209 | 209 | 209 | 209 | 209 | 209 | 209 | 209 | 209 | 209 | 209 | 209 |
| SF36\_vital | Pearson Correlation | -,565(\*\*) | -,605(\*\*) | ,479(\*\*) | ,467(\*\*) | ,473(\*\*) | ,566(\*\*) | 1 | ,617(\*\*) | ,516(\*\*) | ,681(\*\*) | -,476(\*\*) | -,021 | -,467(\*\*) |
| Sig. (2-tailed) | ,000 | ,000 | ,000 | ,000 | ,000 | ,000 |  | ,000 | ,000 | ,000 | ,000 | ,758 | ,000 |
| N | 209 | 209 | 209 | 209 | 209 | 209 | 209 | 209 | 209 | 209 | 209 | 209 | 209 |
| SF36\_lim\_soc | Pearson Correlation | -,635(\*\*) | -,633(\*\*) | ,473(\*\*) | ,474(\*\*) | ,498(\*\*) | ,584(\*\*) | ,617(\*\*) | 1 | ,566(\*\*) | ,618(\*\*) | -,502(\*\*) | -,071 | -,530(\*\*) |
| Sig. (2-tailed) | ,000 | ,000 | ,000 | ,000 | ,000 | ,000 | ,000 |  | ,000 | ,000 | ,000 | ,309 | ,000 |
| N | 209 | 209 | 209 | 209 | 209 | 209 | 209 | 209 | 209 | 209 | 209 | 209 | 209 |
| SF36\_lim\_emo | Pearson Correlation | -,528(\*\*) | -,546(\*\*) | ,368(\*\*) | ,458(\*\*) | ,411(\*\*) | ,412(\*\*) | ,516(\*\*) | ,566(\*\*) | 1 | ,589(\*\*) | -,400(\*\*) | -,185(\*\*) | -,299(\*\*) |
| Sig. (2-tailed) | ,000 | ,000 | ,000 | ,000 | ,000 | ,000 | ,000 | ,000 |  | ,000 | ,000 | ,007 | ,000 |
| N | 209 | 209 | 209 | 209 | 209 | 209 | 209 | 209 | 209 | 209 | 209 | 209 | 209 |
| SF36\_sal\_men | Pearson Correlation | -,662(\*\*) | -,614(\*\*) | ,370(\*\*) | ,327(\*\*) | ,340(\*\*) | ,464(\*\*) | ,681(\*\*) | ,618(\*\*) | ,589(\*\*) | 1 | -,471(\*\*) | -,117 | -,455(\*\*) |
| Sig. (2-tailed) | ,000 | ,000 | ,000 | ,000 | ,000 | ,000 | ,000 | ,000 | ,000 |  | ,000 | ,091 | ,000 |
| N | 209 | 209 | 209 | 209 | 209 | 209 | 209 | 209 | 209 | 209 | 209 | 209 | 209 |
| FATTORE1 | Pearson Correlation | ,565(\*\*) | ,406(\*\*) | -,419(\*\*) | -,401(\*\*) | -,379(\*\*) | -,595(\*\*) | -,476(\*\*) | -,502(\*\*) | -,400(\*\*) | -,471(\*\*) | 1 | ,306(\*\*) | ,680(\*\*) |
| Sig. (2-tailed) | ,000 | ,000 | ,000 | ,000 | ,000 | ,000 | ,000 | ,000 | ,000 | ,000 |  | ,000 | ,000 |
| N | 212 | 212 | 209 | 209 | 209 | 209 | 209 | 209 | 209 | 209 | 212 | 212 | 212 |
| FATTORE 2 | Pearson Correlation | ,110 | ,005 | -,155(\*) | -,122 | -,095 | -,076 | -,021 | -,071 | -,185(\*\*) | -,117 | ,306(\*\*) | 1 | ,170(\*) |
| Sig. (2-tailed) | ,111 | ,941 | ,025 | ,079 | ,173 | ,274 | ,758 | ,309 | ,007 | ,091 | ,000 |  | ,013 |
| N | 212 | 212 | 209 | 209 | 209 | 209 | 209 | 209 | 209 | 209 | 212 | 212 | 212 |
| FATTORE 3 | Pearson Correlation | ,461(\*\*) | ,391(\*\*) | -,468(\*\*) | -,398(\*\*) | -,372(\*\*) | -,561(\*\*) | -,467(\*\*) | -,530(\*\*) | -,299(\*\*) | -,455(\*\*) | ,680(\*\*) | ,170(\*) | 1 |
| Sig. (2-tailed) | ,000 | ,000 | ,000 | ,000 | ,000 | ,000 | ,000 | ,000 | ,000 | ,000 | ,000 | ,013 |  |
| N | 212 | 212 | 209 | 209 | 209 | 209 | 209 | 209 | 209 | 209 | 212 | 212 | 212 |
| \*\* Correlation is significant at the 0.01 level (2-tailed). | | | | | | | | | | | | | | |
| \* Correlation is significant at the 0.05 level (2-tailed). | | | | | | | | | | | | | | |

  
