# Supplementary material for: Cancer-Related Psychological Distress in Lymphoma Survivor: An Italian Cross-Sectional Study
Source: Front Psychol. 2022 Apr 26;13:872329. doi: 10.3389/fpsyg.2022.872329 (PMC9088809; doi:10.3389/fpsyg.2022.872329)
Supplement: Supplementary file 1 [file Data_Sheet_1.zip › STATISTIC ANALYSIS/05_T-Test_A_D-HD_NHL.HTM]

<!--Text used as the document title (displayed in the title bar).-->


# T-Test


Notes

| Output Created | | 26-DEC-2020 10:16:42 |
| Comments | |  |
| Input | Data | C:\Users\Barbara\cro\analisi\_dati\survivors\_linfomi\_dati2020\dati\_2020\_survivor\_linfoma\_n212.sav |
| Filter | <none> |
| Weight | <none> |
| Split File | <none> |
| N of Rows in Working Data File | 212 |
| Missing Value Handling | Definition of Missing | User defined missing values are treated as missing. |
| Cases Used | Statistics for each analysis are based on the cases with no missing or out-of-range data for any variable in the analysis. |
| Syntax | | T-TEST  GROUPS = Diagnosi(1 2)  /MISSING = ANALYSIS  /VARIABLES = a\_hads\_a a\_hads\_d  /CRITERIA = CI(.95) . |
| Resources | Elapsed Time | 0:00:00,06 |

  


Group Statistics

|  | Diagnosi | N | Mean | Std. Deviation | Std. Error Mean |
| a\_hads\_a | 1 | 102 | 6,27 | 3,574 | ,354 |
| 2 | 110 | 5,21 | 3,788 | ,361 |
| a\_hads\_d | 1 | 102 | 4,29 | 3,017 | ,299 |
| 2 | 110 | 3,75 | 2,940 | ,280 |

  


Independent Samples Test

|  |  | Levene's Test for Equality of Variances | | t-test for Equality of Means | | | | | | |
| F | Sig. | t | df | Sig. (2-tailed) | Mean Difference | Std. Error Difference | 95% Confidence Interval of the Difference | |
| Lower | Upper |
| a\_hads\_a | Equal variances assumed | ,359 | ,550 | 2,102 | 210 | ,037 | 1,065 | ,507 | ,066 | 2,064 |
| Equal variances not assumed |  |  | 2,107 | 209,934 | ,036 | 1,065 | ,506 | ,069 | 2,062 |
| a\_hads\_d | Equal variances assumed | ,387 | ,535 | 1,318 | 210 | ,189 | ,540 | ,409 | -,267 | 1,346 |
| Equal variances not assumed |  |  | 1,317 | 207,859 | ,189 | ,540 | ,410 | -,268 | 1,347 |

  
