# Supplementary material for: Cancer-Related Psychological Distress in Lymphoma Survivor: An Italian Cross-Sectional Study
Source: Front Psychol. 2022 Apr 26;13:872329. doi: 10.3389/fpsyg.2022.872329 (PMC9088809; doi:10.3389/fpsyg.2022.872329)
Supplement: Supplementary file 1 [file Data_Sheet_1.zip › STATISTIC ANALYSIS/06B_Crosstabs_D-HD_NHL.HTM]

<!--Text used as the document title (displayed in the title bar).-->


# Crosstabs


Notes

| Output Created | | 26-DEC-2020 10:27:49 |
| Comments | |  |
| Input | Data | C:\Users\Barbara\cro\analisi\_dati\survivors\_linfomi\_dati2020\dati\_2020\_survivor\_linfoma\_n212.sav |
| Filter | <none> |
| Weight | <none> |
| Split File | <none> |
| N of Rows in Working Data File | 212 |
| Missing Value Handling | Definition of Missing | User-defined missing values are treated as missing. |
| Cases Used | Statistics for each table are based on all the cases with valid data in the specified range(s) for all variables in each table. |
| Syntax | | CROSSTABS  /TABLES=Diagnosi BY caseness\_depressione  /FORMAT= AVALUE TABLES  /STATISTIC=CHISQ  /CELLS= COUNT TOTAL  /COUNT ROUND CELL . |
| Resources | Elapsed Time | 0:00:00,03 |
| Dimensions Requested | 2 |
| Cells Available | 116508 |

  


Case Processing Summary

|  | Cases | | | | | |
| Valid | | Missing | | Total | |
| N | Percent | N | Percent | N | Percent |
| Diagnosi \* caseness\_depressione | 212 | 100,0% | 0 | ,0% | 212 | 100,0% |

  


Diagnosi \* caseness\_depressione Crosstabulation

|  |  |  | caseness\_depressione | | Total |
| ,00 | 1,00 |  

| Diagnosi | 1 | Count | 88 | 14 | 102 |
| % of Total | 41,5% | 6,6% | 48,1% |
| 2 | Count | 98 | 12 | 110 |
| % of Total | 46,2% | 5,7% | 51,9% |
| Total | | Count | 186 | 26 | 212 |
| % of Total | 87,7% | 12,3% | 100,0% |

  


Chi-Square Tests

|  | Value | df | Asymp. Sig. (2-sided) | Exact Sig. (2-sided) | Exact Sig. (1-sided) |
| Pearson Chi-Square | ,390(b) | 1 | ,532 |  |  |
| Continuity Correction(a) | ,172 | 1 | ,678 |  |  |
| Likelihood Ratio | ,390 | 1 | ,532 |  |  |
| Fisher's Exact Test |  |  |  | ,539 | ,339 |
| Linear-by-Linear Association | ,388 | 1 | ,533 |  |  |
| N of Valid Cases | 212 |  |  |  |  |
| a Computed only for a 2x2 table | | | | | |
| b 0 cells (,0%) have expected count less than 5. The minimum expected count is 12,51. | | | | | |

  
