# Supplementary material for: Cancer-Related Psychological Distress in Lymphoma Survivor: An Italian Cross-Sectional Study
Source: Front Psychol. 2022 Apr 26;13:872329. doi: 10.3389/fpsyg.2022.872329 (PMC9088809; doi:10.3389/fpsyg.2022.872329)
Supplement: Supplementary file 1 [file Data_Sheet_1.zip › STATISTIC ANALYSIS/07_Correlations_A_D-TIME SURVIVORSHIP.HTM]

<!--Text used as the document title (displayed in the title bar).-->


# Correlations


Notes

| Output Created | | 26-DEC-2020 10:33:20 |
| Comments | |  |
| Input | Data | C:\Users\Barbara\cro\analisi\_dati\survivors\_linfomi\_dati2020\dati\_2020\_survivor\_linfoma\_n212.sav |
| Filter | <none> |
| Weight | <none> |
| Split File | <none> |
| N of Rows in Working Data File | 212 |
| Missing Value Handling | Definition of Missing | User-defined missing values are treated as missing. |
| Cases Used | Statistics for each pair of variables are based on all the cases with valid data for that pair. |
| Syntax | | CORRELATIONS  /VARIABLES=a\_hads\_a a\_hads\_d Annitrascorsidalladiagnosi  /PRINT=TWOTAIL NOSIG  /MISSING=PAIRWISE . |
| Resources | Elapsed Time | 0:00:00,02 |

  


Correlations

|  |  | a\_hads\_a | a\_hads\_d | Anni trascorsi dalla diagnosi |
| a\_hads\_a | Pearson Correlation | 1 | ,711(\*\*) | ,128 |
| Sig. (2-tailed) |  | ,000 | ,063 |
| N | 212 | 212 | 212 |
| a\_hads\_d | Pearson Correlation | ,711(\*\*) | 1 | ,121 |
| Sig. (2-tailed) | ,000 |  | ,079 |
| N | 212 | 212 | 212 |
| Anni trascorsi dalla diagnosi | Pearson Correlation | ,128 | ,121 | 1 |
| Sig. (2-tailed) | ,063 | ,079 |  |
| N | 212 | 212 | 212 |
| \*\* Correlation is significant at the 0.01 level (2-tailed). | | | | |

  
