# Supplementary material for: Cancer-Related Psychological Distress in Lymphoma Survivor: An Italian Cross-Sectional Study
Source: Front Psychol. 2022 Apr 26;13:872329. doi: 10.3389/fpsyg.2022.872329 (PMC9088809; doi:10.3389/fpsyg.2022.872329)
Supplement: Supplementary file 1 [file Data_Sheet_1.zip › STATISTIC ANALYSIS/08_Frequencies_TIME SURVIVORSHIP.HTM]

<!--Text used as the document title (displayed in the title bar).-->


# Frequencies


Notes

| Output Created | | 26-DEC-2020 10:35:30 |
| Comments | |  |
| Input | Data | C:\Users\Barbara\cro\analisi\_dati\survivors\_linfomi\_dati2020\dati\_2020\_survivor\_linfoma\_n212.sav |
| Filter | <none> |
| Weight | <none> |
| Split File | <none> |
| N of Rows in Working Data File | 212 |
| Missing Value Handling | Definition of Missing | User-defined missing values are treated as missing. |
| Cases Used | Statistics are based on all cases with valid data. |
| Syntax | | FREQUENCIES  VARIABLES=Annitrascorsidalladiagnosi  /ORDER= ANALYSIS . |
| Resources | Elapsed Time | 0:00:00,03 |
| Total Values Allowed | 149796 |

  


Statistics
  
 Anni trascorsi dalla diagnosi

| N | Valid | 212 |
| Missing | 0 |

  


Anni trascorsi dalla diagnosi

|  |  | Frequency | Percent | Valid Percent | Cumulative Percent |
| Valid | 5 | 4 | 1,9 | 1,9 | 1,9 |
| 6 | 17 | 8,0 | 8,0 | 9,9 |
| 7 | 17 | 8,0 | 8,0 | 17,9 |
| 8 | 27 | 12,7 | 12,7 | 30,7 |
| 9 | 15 | 7,1 | 7,1 | 37,7 |
| 10 | 14 | 6,6 | 6,6 | 44,3 |
| 11 | 18 | 8,5 | 8,5 | 52,8 |
| 12 | 19 | 9,0 | 9,0 | 61,8 |
| 13 | 7 | 3,3 | 3,3 | 65,1 |
| 14 | 7 | 3,3 | 3,3 | 68,4 |
| 15 | 7 | 3,3 | 3,3 | 71,7 |
| 16 | 9 | 4,2 | 4,2 | 75,9 |
| 17 | 4 | 1,9 | 1,9 | 77,8 |
| 18 | 8 | 3,8 | 3,8 | 81,6 |
| 19 | 2 | ,9 | ,9 | 82,5 |
| 20 | 6 | 2,8 | 2,8 | 85,4 |
| 21 | 4 | 1,9 | 1,9 | 87,3 |
| 22 | 2 | ,9 | ,9 | 88,2 |
| 23 | 6 | 2,8 | 2,8 | 91,0 |
| 24 | 3 | 1,4 | 1,4 | 92,5 |
| 25 | 2 | ,9 | ,9 | 93,4 |
| 26 | 1 | ,5 | ,5 | 93,9 |
| 27 | 1 | ,5 | ,5 | 94,3 |
| 28 | 1 | ,5 | ,5 | 94,8 |
| 30 | 3 | 1,4 | 1,4 | 96,2 |
| 31 | 2 | ,9 | ,9 | 97,2 |
| 32 | 1 | ,5 | ,5 | 97,6 |
| 33 | 1 | ,5 | ,5 | 98,1 |
| 35 | 1 | ,5 | ,5 | 98,6 |
| 36 | 2 | ,9 | ,9 | 99,5 |
| 37 | 1 | ,5 | ,5 | 100,0 |
| Total | 212 | 100,0 | 100,0 |  |

  
