# Supplementary material for: Cancer-Related Psychological Distress in Lymphoma Survivor: An Italian Cross-Sectional Study
Source: Front Psychol. 2022 Apr 26;13:872329. doi: 10.3389/fpsyg.2022.872329 (PMC9088809; doi:10.3389/fpsyg.2022.872329)
Supplement: Supplementary file 1 [file Data_Sheet_1.zip › STATISTIC ANALYSIS/11_T-Test_M_F-A_D.HTM]

<!--Text used as the document title (displayed in the title bar).-->


# T-Test


Notes

| Output Created | | 16-JAN-2021 17:02:59 |
| Comments | |  |
| Input | Data | C:\Users\Barbara\cro\analisi\_dati\survivors\_linfomi\_dati2020\database\_12\_gennaio\_2021\dati\_12\_gennaio\_2021.sav |
| Filter | <none> |
| Weight | <none> |
| Split File | <none> |
| N of Rows in Working Data File | 212 |
| Missing Value Handling | Definition of Missing | User defined missing values are treated as missing. |
| Cases Used | Statistics for each analysis are based on the cases with no missing or out-of-range data for any variable in the analysis. |
| Syntax | | T-TEST  GROUPS = Sesso(1 2)  /MISSING = ANALYSIS  /VARIABLES = a\_hads\_a a\_hads\_d  /CRITERIA = CI(.95) . |
| Resources | Elapsed Time | 0:00:00,06 |

  


Group Statistics

|  | Sesso | N | Mean | Std. Deviation | Std. Error Mean |
| a\_hads\_a | 1 | 117 | 4,85 | 3,392 | ,314 |
| 2 | 95 | 6,79 | 3,837 | ,394 |
| a\_hads\_d | 1 | 117 | 3,61 | 2,813 | ,260 |
| 2 | 95 | 4,52 | 3,121 | ,320 |

  


Independent Samples Test

|  |  | Levene's Test for Equality of Variances | | t-test for Equality of Means | | | | | | |
| F | Sig. | t | df | Sig. (2-tailed) | Mean Difference | Std. Error Difference | 95% Confidence Interval of the Difference | |
| Lower | Upper |
| a\_hads\_a | Equal variances assumed | 2,428 | ,121 | -3,894 | 210 | ,000 | -1,935 | ,497 | -2,914 | -,955 |
| Equal variances not assumed |  |  | -3,844 | 189,359 | ,000 | -1,935 | ,503 | -2,928 | -,942 |
| a\_hads\_d | Equal variances assumed | 3,884 | ,050 | -2,227 | 210 | ,027 | -,909 | ,408 | -1,713 | -,104 |
| Equal variances not assumed |  |  | -2,203 | 191,412 | ,029 | -,909 | ,413 | -1,723 | -,095 |

  
