# Supplementary material for: Cancer-Related Psychological Distress in Lymphoma Survivor: An Italian Cross-Sectional Study
Source: Front Psychol. 2022 Apr 26;13:872329. doi: 10.3389/fpsyg.2022.872329 (PMC9088809; doi:10.3389/fpsyg.2022.872329)
Supplement: Supplementary file 1 [file Data_Sheet_1.zip › STATISTIC ANALYSIS/12_Oneway_AGE AT SURVEY-A_D.HTM]

<!--Text used as the document title (displayed in the title bar).-->


# Oneway


Notes

| Output Created | | 16-JAN-2021 17:12:56 |
| Comments | |  |
| Input | Data | C:\Users\Barbara\cro\analisi\_dati\survivors\_linfomi\_dati2020\database\_12\_gennaio\_2021\dati\_12\_gennaio\_2021.sav |
| Filter | <none> |
| Weight | <none> |
| Split File | <none> |
| N of Rows in Working Data File | 212 |
| Missing Value Handling | Definition of Missing | User-defined missing values are treated as missing. |
| Cases Used | Statistics for each analysis are based on cases with no missing data for any variable in the analysis. |
| Syntax | | ONEWAY  a\_hads\_a a\_hads\_d BY eta\_4\_cat  /STATISTICS DESCRIPTIVES  /MISSING ANALYSIS . |
| Resources | Elapsed Time | 0:00:00,06 |

  


Descriptives

|  |  | N | Mean | Std. Deviation | Std. Error | 95% Confidence Interval for Mean | | Minimum | Maximum |
| Lower Bound | Upper Bound |  
  

| a\_hads\_a | 1 | 9 | 4,78 | 3,528 | 1,176 | 2,07 | 7,49 | 0 | 11 |
| 2 | 99 | 6,33 | 3,662 | ,368 | 5,60 | 7,06 | 0 | 18 |
| 3 | 85 | 5,29 | 3,741 | ,406 | 4,49 | 6,10 | 0 | 15 |
| 4 | 19 | 4,89 | 3,740 | ,858 | 3,09 | 6,70 | 0 | 12 |
| Total | 212 | 5,72 | 3,717 | ,255 | 5,22 | 6,22 | 0 | 18 |
| a\_hads\_d | 1 | 9 | 4,11 | 3,621 | 1,207 | 1,33 | 6,89 | 1 | 11 |
| 2 | 99 | 3,73 | 2,788 | ,280 | 3,17 | 4,28 | 0 | 13 |
| 3 | 85 | 4,32 | 3,174 | ,344 | 3,63 | 5,00 | 0 | 16 |
| 4 | 19 | 4,11 | 2,865 | ,657 | 2,72 | 5,49 | 0 | 11 |
| Total | 212 | 4,01 | 2,983 | ,205 | 3,61 | 4,42 | 0 | 16 |

  


ANOVA

|  |  | Sum of Squares | df | Mean Square | F | Sig. |
| a\_hads\_a | Between Groups | 73,588 | 3 | 24,529 | 1,796 | ,149 |
| Within Groups | 2840,992 | 208 | 13,659 |  |  |
| Total | 2914,580 | 211 |  |  |  |
| a\_hads\_d | Between Groups | 16,219 | 3 | 5,406 | ,604 | ,613 |
| Within Groups | 1860,738 | 208 | 8,946 |  |  |
| Total | 1876,958 | 211 |  |  |  |

  
