# Supplementary material for: Cancer-Related Psychological Distress in Lymphoma Survivor: An Italian Cross-Sectional Study
Source: Front Psychol. 2022 Apr 26;13:872329. doi: 10.3389/fpsyg.2022.872329 (PMC9088809; doi:10.3389/fpsyg.2022.872329)
Supplement: Supplementary file 1 [file Data_Sheet_1.zip › STATISTIC ANALYSIS/14_Oneway_EDUCATIONAL LEVEL-A_D.HTM]

<!--Text used as the document title (displayed in the title bar).-->


# Oneway


Notes

| Output Created | | 16-JAN-2021 17:22:21 |
| Comments | |  |
| Input | Data | C:\Users\Barbara\cro\analisi\_dati\survivors\_linfomi\_dati2020\database\_12\_gennaio\_2021\dati\_12\_gennaio\_2021.sav |
| Filter | <none> |
| Weight | <none> |
| Split File | <none> |
| N of Rows in Working Data File | 212 |
| Missing Value Handling | Definition of Missing | User-defined missing values are treated as missing. |
| Cases Used | Statistics for each analysis are based on cases with no missing data for any variable in the analysis. |
| Syntax | | ONEWAY  a\_hads\_a a\_hads\_d BY education\_5\_cat  /STATISTICS DESCRIPTIVES  /MISSING ANALYSIS . |
| Resources | Elapsed Time | 0:00:00,04 |

  


Descriptives

|  |  | N | Mean | Std. Deviation | Std. Error | 95% Confidence Interval for Mean | | Minimum | Maximum |
| Lower Bound | Upper Bound |  
  

| a\_hads\_a | 1 | 31 | 4,81 | 3,027 | ,544 | 3,70 | 5,92 | 0 | 11 |
| 2 | 62 | 6,05 | 4,444 | ,564 | 4,92 | 7,18 | 0 | 18 |
| 3 | 80 | 6,06 | 3,534 | ,395 | 5,28 | 6,85 | 0 | 16 |
| 4 | 39 | 5,23 | 3,232 | ,518 | 4,18 | 6,28 | 0 | 16 |
| Total | 212 | 5,72 | 3,717 | ,255 | 5,22 | 6,22 | 0 | 18 |
| a\_hads\_d | 1 | 31 | 4,03 | 2,811 | ,505 | 3,00 | 5,06 | 0 | 11 |
| 2 | 62 | 4,15 | 2,936 | ,373 | 3,40 | 4,89 | 1 | 15 |
| 3 | 80 | 3,96 | 3,164 | ,354 | 3,26 | 4,67 | 0 | 16 |
| 4 | 39 | 3,90 | 2,909 | ,466 | 2,95 | 4,84 | 0 | 11 |
| Total | 212 | 4,01 | 2,983 | ,205 | 3,61 | 4,42 | 0 | 16 |

  


ANOVA

|  |  | Sum of Squares | df | Mean Square | F | Sig. |
| a\_hads\_a | Between Groups | 51,276 | 3 | 17,092 | 1,242 | ,296 |
| Within Groups | 2863,304 | 208 | 13,766 |  |  |
| Total | 2914,580 | 211 |  |  |  |
| a\_hads\_d | Between Groups | 1,819 | 3 | ,606 | ,067 | ,977 |
| Within Groups | 1875,139 | 208 | 9,015 |  |  |
| Total | 1876,958 | 211 |  |  |  |

  
