# Supplementary material for: Cancer-Related Psychological Distress in Lymphoma Survivor: An Italian Cross-Sectional Study
Source: Front Psychol. 2022 Apr 26;13:872329. doi: 10.3389/fpsyg.2022.872329 (PMC9088809; doi:10.3389/fpsyg.2022.872329)
Supplement: Supplementary file 1 [file Data_Sheet_1.zip › STATISTIC ANALYSIS/15_T-Test_EMPLOYMENT STATUS-A_D.HTM]

<!--Text used as the document title (displayed in the title bar).-->


# T-Test


Notes

| Output Created | | 16-JAN-2021 17:26:41 |
| Comments | |  |
| Input | Data | C:\Users\Barbara\cro\analisi\_dati\survivors\_linfomi\_dati2020\database\_12\_gennaio\_2021\dati\_12\_gennaio\_2021.sav |
| Filter | <none> |
| Weight | <none> |
| Split File | <none> |
| N of Rows in Working Data File | 212 |
| Missing Value Handling | Definition of Missing | User defined missing values are treated as missing. |
| Cases Used | Statistics for each analysis are based on the cases with no missing or out-of-range data for any variable in the analysis. |
| Syntax | | T-TEST  GROUPS = employment\_dic(1 2)  /MISSING = ANALYSIS  /VARIABLES = a\_hads\_a a\_hads\_d  /CRITERIA = CI(.95) . |
| Resources | Elapsed Time | 0:00:00,22 |

  


Group Statistics

|  | employment\_dic | N | Mean | Std. Deviation | Std. Error Mean |
| a\_hads\_a | 1 | 117 | 5,67 | 3,291 | ,304 |
| 2 | 95 | 5,79 | 4,200 | ,431 |
| a\_hads\_d | 1 | 117 | 3,68 | 2,760 | ,255 |
| 2 | 95 | 4,43 | 3,201 | ,328 |

  


Independent Samples Test

|  |  | Levene's Test for Equality of Variances | | t-test for Equality of Means | | | | | | |
| F | Sig. | t | df | Sig. (2-tailed) | Mean Difference | Std. Error Difference | 95% Confidence Interval of the Difference | |
| Lower | Upper |
| a\_hads\_a | Equal variances assumed | 7,318 | ,007 | -,239 | 210 | ,812 | -,123 | ,514 | -1,137 | ,891 |
| Equal variances not assumed |  |  | -,233 | 175,697 | ,816 | -,123 | ,527 | -1,164 | ,918 |
| a\_hads\_d | Equal variances assumed | 1,002 | ,318 | -1,847 | 210 | ,066 | -,756 | ,410 | -1,564 | ,051 |
| Equal variances not assumed |  |  | -1,819 | 186,623 | ,071 | -,756 | ,416 | -1,577 | ,064 |

  
