# Supplementary material for: Cancer-Related Psychological Distress in Lymphoma Survivor: An Italian Cross-Sectional Study
Source: Front Psychol. 2022 Apr 26;13:872329. doi: 10.3389/fpsyg.2022.872329 (PMC9088809; doi:10.3389/fpsyg.2022.872329)
Supplement: Supplementary file 1 [file Data_Sheet_1.zip › STATISTIC ANALYSIS/18A_POST_HOC_T-Test_PHYSICAL ACTIVITY-A.HTM]

<!--Text used as the document title (displayed in the title bar).-->


# T-Test


Notes

| Output Created | | 16-JAN-2021 17:57:40 |
| Comments | |  |
| Input | Data | C:\Users\Barbara\cro\analisi\_dati\survivors\_linfomi\_dati2020\database\_12\_gennaio\_2021\dati\_12\_gennaio\_2021.sav |
| Filter | <none> |
| Weight | <none> |
| Split File | <none> |
| N of Rows in Working Data File | 212 |
| Missing Value Handling | Definition of Missing | User defined missing values are treated as missing. |
| Cases Used | Statistics for each analysis are based on the cases with no missing or out-of-range data for any variable in the analysis. |
| Syntax | | T-TEST  GROUPS = Attivit�Fisica(1 2)  /MISSING = ANALYSIS  /VARIABLES = a\_hads\_a  /CRITERIA = CI(.95) . |
| Resources | Elapsed Time | 0:00:00,04 |

  


Group Statistics

|  | Attivit�-Fisica | N | Mean | Std. Deviation | Std. Error Mean |
| a\_hads\_a | 1 | 97 | 5,16 | 3,334 | ,339 |
| 2 | 64 | 5,41 | 3,853 | ,482 |

  


Independent Samples Test

|  |  | Levene's Test for Equality of Variances | | t-test for Equality of Means | | | | | | |
| F | Sig. | t | df | Sig. (2-tailed) | Mean Difference | Std. Error Difference | 95% Confidence Interval of the Difference | |
| Lower | Upper |
| a\_hads\_a | Equal variances assumed | 1,926 | ,167 | -,422 | 159 | ,673 | -,241 | ,572 | -1,370 | ,888 |
| Equal variances not assumed |  |  | -,410 | 121,212 | ,683 | -,241 | ,589 | -1,407 | ,924 |

  


# T-Test


Notes

| Output Created | | 16-JAN-2021 17:57:41 |
| Comments | |  |
| Input | Data | C:\Users\Barbara\cro\analisi\_dati\survivors\_linfomi\_dati2020\database\_12\_gennaio\_2021\dati\_12\_gennaio\_2021.sav |
| Filter | <none> |
| Weight | <none> |
| Split File | <none> |
| N of Rows in Working Data File | 212 |
| Missing Value Handling | Definition of Missing | User defined missing values are treated as missing. |
| Cases Used | Statistics for each analysis are based on the cases with no missing or out-of-range data for any variable in the analysis. |
| Syntax | | T-TEST  GROUPS = Attivit�Fisica(1 3)  /MISSING = ANALYSIS  /VARIABLES = a\_hads\_a  /CRITERIA = CI(.95) . |
| Resources | Elapsed Time | 0:00:00,04 |

  


Group Statistics

|  | Attivit�-Fisica | N | Mean | Std. Deviation | Std. Error Mean |
| a\_hads\_a | 1 | 97 | 5,16 | 3,334 | ,339 |
| 3 | 51 | 7,18 | 3,918 | ,549 |

  


Independent Samples Test

|  |  | Levene's Test for Equality of Variances | | t-test for Equality of Means | | | | | | |
| F | Sig. | t | df | Sig. (2-tailed) | Mean Difference | Std. Error Difference | 95% Confidence Interval of the Difference | |
| Lower | Upper |
| a\_hads\_a | Equal variances assumed | 3,188 | ,076 | -3,281 | 146 | ,001 | -2,012 | ,613 | -3,223 | -,800 |
| Equal variances not assumed |  |  | -3,120 | 88,643 | ,002 | -2,012 | ,645 | -3,292 | -,731 |

  


# T-Test


Notes

| Output Created | | 16-JAN-2021 17:57:41 |
| Comments | |  |
| Input | Data | C:\Users\Barbara\cro\analisi\_dati\survivors\_linfomi\_dati2020\database\_12\_gennaio\_2021\dati\_12\_gennaio\_2021.sav |
| Filter | <none> |
| Weight | <none> |
| Split File | <none> |
| N of Rows in Working Data File | 212 |
| Missing Value Handling | Definition of Missing | User defined missing values are treated as missing. |
| Cases Used | Statistics for each analysis are based on the cases with no missing or out-of-range data for any variable in the analysis. |
| Syntax | | T-TEST  GROUPS = Attivit�Fisica(2 3)  /MISSING = ANALYSIS  /VARIABLES = a\_hads\_a  /CRITERIA = CI(.95) . |
| Resources | Elapsed Time | 0:00:00,04 |

  


Group Statistics

|  | Attivit�-Fisica | N | Mean | Std. Deviation | Std. Error Mean |
| a\_hads\_a | 2 | 64 | 5,41 | 3,853 | ,482 |
| 3 | 51 | 7,18 | 3,918 | ,549 |

  


Independent Samples Test

|  |  | Levene's Test for Equality of Variances | | t-test for Equality of Means | | | | | | |
| F | Sig. | t | df | Sig. (2-tailed) | Mean Difference | Std. Error Difference | 95% Confidence Interval of the Difference | |
| Lower | Upper |
| a\_hads\_a | Equal variances assumed | ,159 | ,690 | -2,429 | 113 | ,017 | -1,770 | ,729 | -3,214 | -,327 |
| Equal variances not assumed |  |  | -2,425 | 106,550 | ,017 | -1,770 | ,730 | -3,217 | -,323 |

  
