# Supplementary material for: Cancer-Related Psychological Distress in Lymphoma Survivor: An Italian Cross-Sectional Study
Source: Front Psychol. 2022 Apr 26;13:872329. doi: 10.3389/fpsyg.2022.872329 (PMC9088809; doi:10.3389/fpsyg.2022.872329)
Supplement: Supplementary file 1 [file Data_Sheet_1.zip › STATISTIC ANALYSIS/18b_POST_HOC__T-Test_PHYSICAL ACTIVITY-D.HTM]

<!--Text used as the document title (displayed in the title bar).-->


# T-Test


Notes

| Output Created | | 16-JAN-2021 17:50:47 |
| Comments | |  |
| Input | Data | C:\Users\Barbara\cro\analisi\_dati\survivors\_linfomi\_dati2020\database\_12\_gennaio\_2021\dati\_12\_gennaio\_2021.sav |
| Filter | <none> |
| Weight | <none> |
| Split File | <none> |
| N of Rows in Working Data File | 212 |
| Missing Value Handling | Definition of Missing | User defined missing values are treated as missing. |
| Cases Used | Statistics for each analysis are based on the cases with no missing or out-of-range data for any variable in the analysis. |
| Syntax | | T-TEST  GROUPS = Attivit�Fisica(1 2)  /MISSING = ANALYSIS  /VARIABLES = a\_hads\_d  /CRITERIA = CI(.95) . |
| Resources | Elapsed Time | 0:00:00,05 |

  


Group Statistics

|  | Attivit�-Fisica | N | Mean | Std. Deviation | Std. Error Mean |
| a\_hads\_d | 1 | 97 | 3,38 | 2,551 | ,259 |
| 2 | 64 | 4,22 | 2,803 | ,350 |

  


Independent Samples Test

|  |  | Levene's Test for Equality of Variances | | t-test for Equality of Means | | | | | | |
| F | Sig. | t | df | Sig. (2-tailed) | Mean Difference | Std. Error Difference | 95% Confidence Interval of the Difference | |
| Lower | Upper |
| a\_hads\_d | Equal variances assumed | 1,533 | ,217 | -1,959 | 159 | ,052 | -,837 | ,427 | -1,681 | ,007 |
| Equal variances not assumed |  |  | -1,922 | 125,998 | ,057 | -,837 | ,436 | -1,700 | ,025 |

  


# T-Test


Notes

| Output Created | | 16-JAN-2021 17:50:47 |
| Comments | |  |
| Input | Data | C:\Users\Barbara\cro\analisi\_dati\survivors\_linfomi\_dati2020\database\_12\_gennaio\_2021\dati\_12\_gennaio\_2021.sav |
| Filter | <none> |
| Weight | <none> |
| Split File | <none> |
| N of Rows in Working Data File | 212 |
| Missing Value Handling | Definition of Missing | User defined missing values are treated as missing. |
| Cases Used | Statistics for each analysis are based on the cases with no missing or out-of-range data for any variable in the analysis. |
| Syntax | | T-TEST  GROUPS = Attivit�Fisica(1 3)  /MISSING = ANALYSIS  /VARIABLES = a\_hads\_d  /CRITERIA = CI(.95) . |
| Resources | Elapsed Time | 0:00:00,07 |

  


Group Statistics

|  | Attivit�-Fisica | N | Mean | Std. Deviation | Std. Error Mean |
| a\_hads\_d | 1 | 97 | 3,38 | 2,551 | ,259 |
| 3 | 51 | 4,96 | 3,660 | ,513 |

  


Independent Samples Test

|  |  | Levene's Test for Equality of Variances | | t-test for Equality of Means | | | | | | |
| F | Sig. | t | df | Sig. (2-tailed) | Mean Difference | Std. Error Difference | 95% Confidence Interval of the Difference | |
| Lower | Upper |
| a\_hads\_d | Equal variances assumed | 6,150 | ,014 | -3,066 | 146 | ,003 | -1,579 | ,515 | -2,597 | -,561 |
| Equal variances not assumed |  |  | -2,750 | 76,215 | ,007 | -1,579 | ,574 | -2,723 | -,436 |

  


# T-Test


Notes

| Output Created | | 16-JAN-2021 17:50:47 |
| Comments | |  |
| Input | Data | C:\Users\Barbara\cro\analisi\_dati\survivors\_linfomi\_dati2020\database\_12\_gennaio\_2021\dati\_12\_gennaio\_2021.sav |
| Filter | <none> |
| Weight | <none> |
| Split File | <none> |
| N of Rows in Working Data File | 212 |
| Missing Value Handling | Definition of Missing | User defined missing values are treated as missing. |
| Cases Used | Statistics for each analysis are based on the cases with no missing or out-of-range data for any variable in the analysis. |
| Syntax | | T-TEST  GROUPS = Attivit�Fisica(2 3)  /MISSING = ANALYSIS  /VARIABLES = a\_hads\_d  /CRITERIA = CI(.95) . |
| Resources | Elapsed Time | 0:00:00,03 |

  


Group Statistics

|  | Attivit�-Fisica | N | Mean | Std. Deviation | Std. Error Mean |
| a\_hads\_d | 2 | 64 | 4,22 | 2,803 | ,350 |
| 3 | 51 | 4,96 | 3,660 | ,513 |

  


Independent Samples Test

|  |  | Levene's Test for Equality of Variances | | t-test for Equality of Means | | | | | | |
| F | Sig. | t | df | Sig. (2-tailed) | Mean Difference | Std. Error Difference | 95% Confidence Interval of the Difference | |
| Lower | Upper |
| a\_hads\_d | Equal variances assumed | 1,794 | ,183 | -1,231 | 113 | ,221 | -,742 | ,603 | -1,936 | ,452 |
| Equal variances not assumed |  |  | -1,195 | 91,744 | ,235 | -,742 | ,621 | -1,975 | ,491 |

  
