# Supplementary material for: Cancer-Related Psychological Distress in Lymphoma Survivor: An Italian Cross-Sectional Study
Source: Front Psychol. 2022 Apr 26;13:872329. doi: 10.3389/fpsyg.2022.872329 (PMC9088809; doi:10.3389/fpsyg.2022.872329)
Supplement: Supplementary file 1 [file Data_Sheet_1.zip › STATISTIC ANALYSIS/18_Oneway_PHYSICAL ACTIVITY-A_D.HTM]

<!--Text used as the document title (displayed in the title bar).-->


# Oneway


Notes

| Output Created | | 16-JAN-2021 17:39:44 |
| Comments | |  |
| Input | Data | C:\Users\Barbara\cro\analisi\_dati\survivors\_linfomi\_dati2020\database\_12\_gennaio\_2021\dati\_12\_gennaio\_2021.sav |
| Filter | <none> |
| Weight | <none> |
| Split File | <none> |
| N of Rows in Working Data File | 212 |
| Missing Value Handling | Definition of Missing | User-defined missing values are treated as missing. |
| Cases Used | Statistics for each analysis are based on cases with no missing data for any variable in the analysis. |
| Syntax | | ONEWAY  a\_hads\_a a\_hads\_d BY Attivit�Fisica  /STATISTICS DESCRIPTIVES  /MISSING ANALYSIS . |
| Resources | Elapsed Time | 0:00:00,06 |

  


Descriptives

|  |  | N | Mean | Std. Deviation | Std. Error | 95% Confidence Interval for Mean | | Minimum | Maximum |
| Lower Bound | Upper Bound |  
  

| a\_hads\_a | 1 | 97 | 5,16 | 3,334 | ,339 | 4,49 | 5,84 | 0 | 16 |
| 2 | 64 | 5,41 | 3,853 | ,482 | 4,44 | 6,37 | 0 | 15 |
| 3 | 51 | 7,18 | 3,918 | ,549 | 6,07 | 8,28 | 2 | 18 |
| Total | 212 | 5,72 | 3,717 | ,255 | 5,22 | 6,22 | 0 | 18 |
| a\_hads\_d | 1 | 97 | 3,38 | 2,551 | ,259 | 2,87 | 3,90 | 0 | 13 |
| 2 | 64 | 4,22 | 2,803 | ,350 | 3,52 | 4,92 | 0 | 11 |
| 3 | 51 | 4,96 | 3,660 | ,513 | 3,93 | 5,99 | 0 | 16 |
| Total | 212 | 4,01 | 2,983 | ,205 | 3,61 | 4,42 | 0 | 16 |

  


ANOVA

|  |  | Sum of Squares | df | Mean Square | F | Sig. |
| a\_hads\_a | Between Groups | 144,370 | 2 | 72,185 | 5,446 | ,005 |
| Within Groups | 2770,210 | 209 | 13,255 |  |  |
| Total | 2914,580 | 211 |  |  |  |
| a\_hads\_d | Between Groups | 87,212 | 2 | 43,606 | 5,092 | ,007 |
| Within Groups | 1789,746 | 209 | 8,563 |  |  |
| Total | 1876,958 | 211 |  |  |  |

  
