# Supplementary material for: Cancer-Related Psychological Distress in Lymphoma Survivor: An Italian Cross-Sectional Study
Source: Front Psychol. 2022 Apr 26;13:872329. doi: 10.3389/fpsyg.2022.872329 (PMC9088809; doi:10.3389/fpsyg.2022.872329)
Supplement: Supplementary file 1 [file Data_Sheet_1.zip › STATISTIC ANALYSIS/19_T-Test_PSYCHOLOGICAL SUPPORT-A_D.HTM]

<!--Text used as the document title (displayed in the title bar).-->


# T-Test


Notes

| Output Created | | 16-JAN-2021 18:13:13 |
| Comments | |  |
| Input | Data | C:\Users\Barbara\cro\analisi\_dati\survivors\_linfomi\_dati2020\database\_12\_gennaio\_2021\dati\_12\_gennaio\_2021.sav |
| Filter | <none> |
| Weight | <none> |
| Split File | <none> |
| N of Rows in Working Data File | 212 |
| Missing Value Handling | Definition of Missing | User defined missing values are treated as missing. |
| Cases Used | Statistics for each analysis are based on the cases with no missing or out-of-range data for any variable in the analysis. |
| Syntax | | T-TEST  GROUPS = Supporto\_Psic\_Beneficiato(1 2)  /MISSING = ANALYSIS  /VARIABLES = a\_hads\_a a\_hads\_d  /CRITERIA = CI(.95) . |
| Resources | Elapsed Time | 0:00:00,05 |

  


Group Statistics

|  | Supporto\_Psic\_Beneficiato | N | Mean | Std. Deviation | Std. Error Mean |
| a\_hads\_a | 1 | 82 | 5,78 | 3,531 | ,390 |
| 2 | 130 | 5,68 | 3,842 | ,337 |
| a\_hads\_d | 1 | 82 | 4,15 | 3,171 | ,350 |
| 2 | 130 | 3,93 | 2,867 | ,251 |

  


Independent Samples Test

|  |  | Levene's Test for Equality of Variances | | t-test for Equality of Means | | | | | | |
| F | Sig. | t | df | Sig. (2-tailed) | Mean Difference | Std. Error Difference | 95% Confidence Interval of the Difference | |
| Lower | Upper |
| a\_hads\_a | Equal variances assumed | ,446 | ,505 | ,182 | 210 | ,855 | ,096 | ,525 | -,940 | 1,131 |
| Equal variances not assumed |  |  | ,186 | 183,039 | ,853 | ,096 | ,515 | -,921 | 1,113 |
| a\_hads\_d | Equal variances assumed | ,314 | ,576 | ,512 | 210 | ,609 | ,216 | ,421 | -,615 | 1,046 |
| Equal variances not assumed |  |  | ,500 | 159,468 | ,618 | ,216 | ,431 | -,636 | 1,067 |

  
