# Supplementary material for: Cancer-Related Psychological Distress in Lymphoma Survivor: An Italian Cross-Sectional Study
Source: Front Psychol. 2022 Apr 26;13:872329. doi: 10.3389/fpsyg.2022.872329 (PMC9088809; doi:10.3389/fpsyg.2022.872329)
Supplement: Supplementary file 1 [file Data_Sheet_1.zip › STATISTIC ANALYSIS/22_Oneway_AGE AT DIAGNOSIS-A_D.HTM]

<!--Text used as the document title (displayed in the title bar).-->


# Oneway


Notes

| Output Created | | 16-JAN-2021 18:40:47 |
| Comments | |  |
| Input | Data | C:\Users\Barbara\cro\analisi\_dati\survivors\_linfomi\_dati2020\database\_12\_gennaio\_2021\dati\_12\_gennaio\_2021.sav |
| Filter | <none> |
| Weight | <none> |
| Split File | <none> |
| N of Rows in Working Data File | 212 |
| Missing Value Handling | Definition of Missing | User-defined missing values are treated as missing. |
| Cases Used | Statistics for each analysis are based on cases with no missing data for any variable in the analysis. |
| Syntax | | ONEWAY  a\_hads\_a a\_hads\_d BY eta\_dia\_4cat  /STATISTICS DESCRIPTIVES  /MISSING ANALYSIS . |
| Resources | Elapsed Time | 0:00:00,06 |

  


Descriptives

|  |  | N | Mean | Std. Deviation | Std. Error | 95% Confidence Interval for Mean | | Minimum | Maximum |
| Lower Bound | Upper Bound |  
  

| a\_hads\_a | 1 | 46 | 6,30 | 3,794 | ,559 | 5,18 | 7,43 | 0 | 18 |
| 2 | 93 | 5,80 | 3,550 | ,368 | 5,06 | 6,53 | 0 | 16 |
| 3 | 65 | 5,51 | 3,953 | ,490 | 4,53 | 6,49 | 0 | 15 |
| 4 | 8 | 3,25 | 2,375 | ,840 | 1,26 | 5,24 | 0 | 6 |
| Total | 212 | 5,72 | 3,717 | ,255 | 5,22 | 6,22 | 0 | 18 |
| a\_hads\_d | 1 | 46 | 3,98 | 2,902 | ,428 | 3,12 | 4,84 | 0 | 11 |
| 2 | 93 | 3,82 | 2,978 | ,309 | 3,20 | 4,43 | 0 | 16 |
| 3 | 65 | 4,46 | 3,138 | ,389 | 3,68 | 5,24 | 0 | 15 |
| 4 | 8 | 2,88 | 1,885 | ,666 | 1,30 | 4,45 | 0 | 5 |
| Total | 212 | 4,01 | 2,983 | ,205 | 3,61 | 4,42 | 0 | 16 |

  


ANOVA

|  |  | Sum of Squares | df | Mean Square | F | Sig. |
| a\_hads\_a | Between Groups | 67,977 | 3 | 22,659 | 1,656 | ,178 |
| Within Groups | 2846,604 | 208 | 13,686 |  |  |
| Total | 2914,580 | 211 |  |  |  |
| a\_hads\_d | Between Groups | 27,058 | 3 | 9,019 | 1,014 | ,387 |
| Within Groups | 1849,900 | 208 | 8,894 |  |  |
| Total | 1876,958 | 211 |  |  |  |

  
