# Supplementary material for: Cancer-Related Psychological Distress in Lymphoma Survivor: An Italian Cross-Sectional Study
Source: Front Psychol. 2022 Apr 26;13:872329. doi: 10.3389/fpsyg.2022.872329 (PMC9088809; doi:10.3389/fpsyg.2022.872329)
Supplement: Supplementary file 1 [file Data_Sheet_1.zip › STATISTIC ANALYSIS/23_Oneway_TIME SINCE DIAGNOSIS-A_D.HTM]

<!--Text used as the document title (displayed in the title bar).-->


# Oneway


Notes

| Output Created | | 16-JAN-2021 18:44:15 |
| Comments | |  |
| Input | Data | C:\Users\Barbara\cro\analisi\_dati\survivors\_linfomi\_dati2020\database\_12\_gennaio\_2021\dati\_12\_gennaio\_2021.sav |
| Filter | <none> |
| Weight | <none> |
| Split File | <none> |
| N of Rows in Working Data File | 212 |
| Missing Value Handling | Definition of Missing | User-defined missing values are treated as missing. |
| Cases Used | Statistics for each analysis are based on cases with no missing data for any variable in the analysis. |
| Syntax | | ONEWAY  a\_hads\_a a\_hads\_d BY surviv\_4cat  /STATISTICS DESCRIPTIVES  /MISSING ANALYSIS . |
| Resources | Elapsed Time | 0:00:00,06 |

  


Descriptives

|  |  | N | Mean | Std. Deviation | Std. Error | 95% Confidence Interval for Mean | | Minimum | Maximum |
| Lower Bound | Upper Bound |  
  

| a\_hads\_a | 1 | 80 | 5,11 | 3,558 | ,398 | 4,32 | 5,90 | 0 | 16 |
| 2 | 65 | 5,58 | 3,464 | ,430 | 4,73 | 6,44 | 0 | 16 |
| 3 | 30 | 6,87 | 3,857 | ,704 | 5,43 | 8,31 | 2 | 14 |
| 4 | 37 | 6,35 | 4,185 | ,688 | 4,96 | 7,75 | 0 | 18 |
| Total | 212 | 5,72 | 3,717 | ,255 | 5,22 | 6,22 | 0 | 18 |
| a\_hads\_d | 1 | 80 | 3,70 | 2,875 | ,321 | 3,06 | 4,34 | 0 | 12 |
| 2 | 65 | 3,86 | 3,061 | ,380 | 3,10 | 4,62 | 0 | 15 |
| 3 | 30 | 4,33 | 2,695 | ,492 | 3,33 | 5,34 | 1 | 10 |
| 4 | 37 | 4,70 | 3,265 | ,537 | 3,61 | 5,79 | 0 | 16 |
| Total | 212 | 4,01 | 2,983 | ,205 | 3,61 | 4,42 | 0 | 16 |

  


ANOVA

|  |  | Sum of Squares | df | Mean Square | F | Sig. |
| a\_hads\_a | Between Groups | 84,909 | 3 | 28,303 | 2,080 | ,104 |
| Within Groups | 2829,671 | 208 | 13,604 |  |  |
| Total | 2914,580 | 211 |  |  |  |
| a\_hads\_d | Between Groups | 30,007 | 3 | 10,002 | 1,126 | ,339 |
| Within Groups | 1846,950 | 208 | 8,880 |  |  |
| Total | 1876,958 | 211 |  |  |  |

  
