# Supplementary material for: Cancer-Related Psychological Distress in Lymphoma Survivor: An Italian Cross-Sectional Study
Source: Front Psychol. 2022 Apr 26;13:872329. doi: 10.3389/fpsyg.2022.872329 (PMC9088809; doi:10.3389/fpsyg.2022.872329)
Supplement: Supplementary file 1 [file Data_Sheet_1.zip › STATISTIC ANALYSIS/24A_T-Test_LIMITED SURGERY.HTM]

<!--Text used as the document title (displayed in the title bar).-->


# T-Test


Notes

| Output Created | | 16-JAN-2021 18:48:18 |
| Comments | |  |
| Input | Data | C:\Users\Barbara\cro\analisi\_dati\survivors\_linfomi\_dati2020\database\_12\_gennaio\_2021\dati\_12\_gennaio\_2021.sav |
| Filter | <none> |
| Weight | <none> |
| Split File | <none> |
| N of Rows in Working Data File | 212 |
| Missing Value Handling | Definition of Missing | User defined missing values are treated as missing. |
| Cases Used | Statistics for each analysis are based on the cases with no missing or out-of-range data for any variable in the analysis. |
| Syntax | | T-TEST  GROUPS = Limitedsurgery(1 2)  /MISSING = ANALYSIS  /VARIABLES = a\_hads\_a a\_hads\_d  /CRITERIA = CI(.95) . |
| Resources | Elapsed Time | 0:00:00,04 |

  


Group Statistics

|  | Limited surgery | N | Mean | Std. Deviation | Std. Error Mean |
| a\_hads\_a | 1 | 207 | 5,76 | 3,727 | ,259 |
| 2 | 5 | 4,20 | 3,194 | 1,428 |
| a\_hads\_d | 1 | 207 | 4,03 | 3,001 | ,209 |
| 2 | 5 | 3,20 | 2,168 | ,970 |

  


Independent Samples Test

|  |  | Levene's Test for Equality of Variances | | t-test for Equality of Means | | | | | | |
| F | Sig. | t | df | Sig. (2-tailed) | Mean Difference | Std. Error Difference | 95% Confidence Interval of the Difference | |
| Lower | Upper |
| a\_hads\_a | Equal variances assumed | ,140 | ,708 | ,926 | 210 | ,355 | 1,558 | 1,683 | -1,759 | 4,875 |
| Equal variances not assumed |  |  | 1,074 | 4,267 | ,340 | 1,558 | 1,452 | -2,374 | 5,491 |
| a\_hads\_d | Equal variances assumed | ,361 | ,549 | ,617 | 210 | ,538 | ,834 | 1,352 | -1,831 | 3,499 |
| Equal variances not assumed |  |  | ,841 | 4,379 | ,444 | ,834 | ,992 | -1,828 | 3,496 |

  
