# Supplementary material for: Cancer-Related Psychological Distress in Lymphoma Survivor: An Italian Cross-Sectional Study
Source: Front Psychol. 2022 Apr 26;13:872329. doi: 10.3389/fpsyg.2022.872329 (PMC9088809; doi:10.3389/fpsyg.2022.872329)
Supplement: Supplementary file 1 [file Data_Sheet_1.zip › STATISTIC ANALYSIS/24B_T-Test_SYSTEMATIC TREATMENT ALONE.HTM]

<!--Text used as the document title (displayed in the title bar).-->


# T-Test


Notes

| Output Created | | 16-JAN-2021 18:51:05 |
| Comments | |  |
| Input | Data | C:\Users\Barbara\cro\analisi\_dati\survivors\_linfomi\_dati2020\database\_12\_gennaio\_2021\dati\_12\_gennaio\_2021.sav |
| Filter | <none> |
| Weight | <none> |
| Split File | <none> |
| N of Rows in Working Data File | 212 |
| Missing Value Handling | Definition of Missing | User defined missing values are treated as missing. |
| Cases Used | Statistics for each analysis are based on the cases with no missing or out-of-range data for any variable in the analysis. |
| Syntax | | T-TEST  GROUPS = Systemictreatmentalone(1 2)  /MISSING = ANALYSIS  /VARIABLES = a\_hads\_a a\_hads\_d  /CRITERIA = CI(.95) . |
| Resources | Elapsed Time | 0:00:00,05 |

  


Group Statistics

|  | Systemic treatment alone | N | Mean | Std. Deviation | Std. Error Mean |
| a\_hads\_a | 1 | 166 | 5,96 | 3,830 | ,297 |
| 2 | 46 | 4,87 | 3,167 | ,467 |
| a\_hads\_d | 1 | 166 | 4,16 | 3,066 | ,238 |
| 2 | 46 | 3,48 | 2,622 | ,387 |

  


Independent Samples Test

|  |  | Levene's Test for Equality of Variances | | t-test for Equality of Means | | | | | | |
| F | Sig. | t | df | Sig. (2-tailed) | Mean Difference | Std. Error Difference | 95% Confidence Interval of the Difference | |
| Lower | Upper |
| a\_hads\_a | Equal variances assumed | 2,194 | ,140 | 1,766 | 210 | ,079 | 1,088 | ,616 | -,126 | 2,303 |
| Equal variances not assumed |  |  | 1,966 | 85,074 | ,053 | 1,088 | ,553 | -,012 | 2,189 |
| a\_hads\_d | Equal variances assumed | 2,739 | ,099 | 1,380 | 210 | ,169 | ,684 | ,496 | -,293 | 1,662 |
| Equal variances not assumed |  |  | 1,507 | 82,313 | ,136 | ,684 | ,454 | -,219 | 1,587 |

  
