# Supplementary material for: Cancer-Related Psychological Distress in Lymphoma Survivor: An Italian Cross-Sectional Study
Source: Front Psychol. 2022 Apr 26;13:872329. doi: 10.3389/fpsyg.2022.872329 (PMC9088809; doi:10.3389/fpsyg.2022.872329)
Supplement: Supplementary file 1 [file Data_Sheet_1.zip › STATISTIC ANALYSIS/24C_T-Test_SYSTEMATIC TREATMENT AND-OR RT.HTM]

<!--Text used as the document title (displayed in the title bar).-->


# T-Test


Notes

| Output Created | | 16-JAN-2021 18:53:20 |
| Comments | |  |
| Input | Data | C:\Users\Barbara\cro\analisi\_dati\survivors\_linfomi\_dati2020\database\_12\_gennaio\_2021\dati\_12\_gennaio\_2021.sav |
| Filter | <none> |
| Weight | <none> |
| Split File | <none> |
| N of Rows in Working Data File | 212 |
| Missing Value Handling | Definition of Missing | User defined missing values are treated as missing. |
| Cases Used | Statistics for each analysis are based on the cases with no missing or out-of-range data for any variable in the analysis. |
| Syntax | | T-TEST  GROUPS = Systemictreatmentandorradiotherapy(1 2)  /MISSING = ANALYSIS  /VARIABLES = a\_hads\_a a\_hads\_d  /CRITERIA = CI(.95) . |
| Resources | Elapsed Time | 0:00:00,05 |

  


Group Statistics

|  | Systemic treatment and/or radiotherapy | N | Mean | Std. Deviation | Std. Error Mean |
| a\_hads\_a | 1 | 71 | 4,61 | 3,200 | ,380 |
| 2 | 141 | 6,28 | 3,840 | ,323 |
| a\_hads\_d | 1 | 71 | 3,34 | 2,443 | ,290 |
| 2 | 141 | 4,35 | 3,174 | ,267 |

  


Independent Samples Test

|  |  | Levene's Test for Equality of Variances | | t-test for Equality of Means | | | | | | |
| F | Sig. | t | df | Sig. (2-tailed) | Mean Difference | Std. Error Difference | 95% Confidence Interval of the Difference | |
| Lower | Upper |
| a\_hads\_a | Equal variances assumed | 2,760 | ,098 | -3,168 | 210 | ,002 | -1,678 | ,530 | -2,722 | -,634 |
| Equal variances not assumed |  |  | -3,364 | 164,948 | ,001 | -1,678 | ,499 | -2,663 | -,693 |
| a\_hads\_d | Equal variances assumed | 7,466 | ,007 | -2,368 | 210 | ,019 | -1,017 | ,429 | -1,863 | -,170 |
| Equal variances not assumed |  |  | -2,578 | 175,970 | ,011 | -1,017 | ,394 | -1,795 | -,238 |

  
