# Supplementary material for: Cancer-Related Psychological Distress in Lymphoma Survivor: An Italian Cross-Sectional Study
Source: Front Psychol. 2022 Apr 26;13:872329. doi: 10.3389/fpsyg.2022.872329 (PMC9088809; doi:10.3389/fpsyg.2022.872329)
Supplement: Supplementary file 1 [file Data_Sheet_1.zip › STATISTIC ANALYSIS/24E_T-Test_TRANSPLANT AND-OR SYSTEMATIC TREATMENT AND-OR RT.HTM]

<!--Text used as the document title (displayed in the title bar).-->


# T-Test


Notes

| Output Created | | 16-JAN-2021 18:58:24 |
| Comments | |  |
| Input | Data | C:\Users\Barbara\cro\analisi\_dati\survivors\_linfomi\_dati2020\database\_12\_gennaio\_2021\dati\_12\_gennaio\_2021.sav |
| Filter | <none> |
| Weight | <none> |
| Split File | <none> |
| N of Rows in Working Data File | 212 |
| Missing Value Handling | Definition of Missing | User defined missing values are treated as missing. |
| Cases Used | Statistics for each analysis are based on the cases with no missing or out-of-range data for any variable in the analysis. |
| Syntax | | T-TEST  GROUPS = TransplantandorSystemictreatmentandorradiotherapy(1 2)  /MISSING = ANALYSIS  /VARIABLES = a\_hads\_a a\_hads\_d  /CRITERIA = CI(.95) . |
| Resources | Elapsed Time | 0:00:00,05 |

  


Group Statistics

|  | Transplant and/or Systemic treatment and/or radiotherapy | N | Mean | Std. Deviation | Std. Error Mean |
| a\_hads\_a | 1 | 208 | 5,75 | 3,724 | ,258 |
| 2 | 4 | 4,25 | 3,403 | 1,702 |
| a\_hads\_d | 1 | 208 | 4,03 | 2,998 | ,208 |
| 2 | 4 | 3,00 | 2,000 | 1,000 |

  


Independent Samples Test

|  |  | Levene's Test for Equality of Variances | | t-test for Equality of Means | | | | | | |
| F | Sig. | t | df | Sig. (2-tailed) | Mean Difference | Std. Error Difference | 95% Confidence Interval of the Difference | |
| Lower | Upper |
| a\_hads\_a | Equal variances assumed | ,232 | ,631 | ,799 | 210 | ,425 | 1,500 | 1,878 | -2,202 | 5,202 |
| Equal variances not assumed |  |  | ,871 | 3,140 | ,445 | 1,500 | 1,721 | -3,842 | 6,842 |
| a\_hads\_d | Equal variances assumed | ,807 | ,370 | ,686 | 210 | ,494 | 1,034 | 1,507 | -1,938 | 4,005 |
| Equal variances not assumed |  |  | 1,012 | 3,265 | ,381 | 1,034 | 1,021 | -2,073 | 4,140 |

  
