# Supplementary material for: Cancer-Related Psychological Distress in Lymphoma Survivor: An Italian Cross-Sectional Study
Source: Front Psychol. 2022 Apr 26;13:872329. doi: 10.3389/fpsyg.2022.872329 (PMC9088809; doi:10.3389/fpsyg.2022.872329)
Supplement: Supplementary file 1 [file Data_Sheet_1.zip › STATISTIC ANALYSIS/25_T-Test_EATING STYLE-A_D.HTM]

<!--Text used as the document title (displayed in the title bar).-->


# T-Test


Notes

| Output Created | | 22-JAN-2021 18:23:02 |
| Comments | |  |
| Input | Data | C:\Users\Barbara\cro\analisi\_dati\survivors\_linfomi\_dati2020\database\_12\_gennaio\_2021\dati\_12\_gennaio\_2021.sav |
| Filter | <none> |
| Weight | <none> |
| Split File | <none> |
| N of Rows in Working Data File | 212 |
| Missing Value Handling | Definition of Missing | User defined missing values are treated as missing. |
| Cases Used | Statistics for each analysis are based on the cases with no missing or out-of-range data for any variable in the analysis. |
| Syntax | | T-TEST  GROUPS = alimentazione\_2\_cat(1 2)  /MISSING = ANALYSIS  /VARIABLES = a\_hads\_a a\_hads\_d  /CRITERIA = CI(.95) . |
| Resources | Elapsed Time | 0:00:00,05 |

  


Group Statistics

|  | alimentazione\_2\_cat | N | Mean | Std. Deviation | Std. Error Mean |
| a\_hads\_a | 1,00 | 194 | 5,82 | 3,755 | ,270 |
| 2,00 | 18 | 4,67 | 3,181 | ,750 |
| a\_hads\_d | 1,00 | 194 | 4,07 | 3,009 | ,216 |
| 2,00 | 18 | 3,39 | 2,682 | ,632 |

  


Independent Samples Test

|  |  | Levene's Test for Equality of Variances | | t-test for Equality of Means | | | | | | |
| F | Sig. | t | df | Sig. (2-tailed) | Mean Difference | Std. Error Difference | 95% Confidence Interval of the Difference | |
| Lower | Upper |
| a\_hads\_a | Equal variances assumed | ,320 | ,572 | 1,261 | 210 | ,209 | 1,153 | ,914 | -,650 | 2,956 |
| Equal variances not assumed |  |  | 1,447 | 21,648 | ,162 | 1,153 | ,797 | -,501 | 2,807 |
| a\_hads\_d | Equal variances assumed | ,365 | ,546 | ,929 | 210 | ,354 | ,683 | ,735 | -,766 | 2,132 |
| Equal variances not assumed |  |  | 1,023 | 21,176 | ,318 | ,683 | ,668 | -,705 | 2,072 |

  
