# Supplementary material for: Cancer-Related Psychological Distress in Lymphoma Survivor: An Italian Cross-Sectional Study
Source: Front Psychol. 2022 Apr 26;13:872329. doi: 10.3389/fpsyg.2022.872329 (PMC9088809; doi:10.3389/fpsyg.2022.872329)
Supplement: Supplementary file 1 [file Data_Sheet_1.zip › STATISTIC ANALYSIS/26A_Crosstabs_EATING STYLE-A.HTM]

<!--Text used as the document title (displayed in the title bar).-->


# Crosstabs


Notes

| Output Created | | 22-JAN-2021 18:37:46 |
| Comments | |  |
| Input | Data | C:\Users\Barbara\cro\analisi\_dati\survivors\_linfomi\_dati2020\database\_12\_gennaio\_2021\dati\_12\_gennaio\_2021.sav |
| Filter | <none> |
| Weight | <none> |
| Split File | <none> |
| N of Rows in Working Data File | 212 |
| Missing Value Handling | Definition of Missing | User-defined missing values are treated as missing. |
| Cases Used | Statistics for each table are based on all the cases with valid data in the specified range(s) for all variables in each table. |
| Syntax | | CROSSTABS  /TABLES=caseness\_ansia BY alimentazione\_2\_cat  /FORMAT= AVALUE TABLES  /STATISTIC=CHISQ  /CELLS= COUNT TOTAL  /COUNT ROUND CELL . |
| Resources | Elapsed Time | 0:00:00,04 |
| Dimensions Requested | 2 |
| Cells Available | 116508 |

  


Case Processing Summary

|  | Cases | | | | | |
| Valid | | Missing | | Total | |
| N | Percent | N | Percent | N | Percent |
| caseness\_ansia \* alimentazione\_2\_cat | 212 | 100,0% | 0 | ,0% | 212 | 100,0% |

  


caseness\_ansia \* alimentazione\_2\_cat Crosstabulation

|  |  |  | alimentazione\_2\_cat | | Total |
| 1,00 | 2,00 |  

| caseness\_ansia | ,00 | Count | 160 | 16 | 176 |
| % of Total | 75,5% | 7,5% | 83,0% |
| 1,00 | Count | 34 | 2 | 36 |
| % of Total | 16,0% | ,9% | 17,0% |
| Total | | Count | 194 | 18 | 212 |
| % of Total | 91,5% | 8,5% | 100,0% |

  


Chi-Square Tests

|  | Value | df | Asymp. Sig. (2-sided) | Exact Sig. (2-sided) | Exact Sig. (1-sided) |
| Pearson Chi-Square | ,481(b) | 1 | ,488 |  |  |
| Continuity Correction(a) | ,133 | 1 | ,715 |  |  |
| Likelihood Ratio | ,530 | 1 | ,467 |  |  |
| Fisher's Exact Test |  |  |  | ,744 | ,379 |
| Linear-by-Linear Association | ,479 | 1 | ,489 |  |  |
| N of Valid Cases | 212 |  |  |  |  |
| a Computed only for a 2x2 table | | | | | |
| b 1 cells (25,0%) have expected count less than 5. The minimum expected count is 3,06. | | | | | |

  
