# Supplementary material for: Cancer-Related Psychological Distress in Lymphoma Survivor: An Italian Cross-Sectional Study
Source: Front Psychol. 2022 Apr 26;13:872329. doi: 10.3389/fpsyg.2022.872329 (PMC9088809; doi:10.3389/fpsyg.2022.872329)
Supplement: Supplementary file 1 [file Data_Sheet_1.zip › STATISTIC ANALYSIS/27A_POST_HOC_T-Test_SLEEP-A.HTM]

<!--Text used as the document title (displayed in the title bar).-->


# T-Test


Notes

| Output Created | | 22-JAN-2021 18:56:23 |
| Comments | |  |
| Input | Data | C:\Users\Barbara\cro\analisi\_dati\survivors\_linfomi\_dati2020\database\_12\_gennaio\_2021\dati\_12\_gennaio\_2021.sav |
| Filter | <none> |
| Weight | <none> |
| Split File | <none> |
| N of Rows in Working Data File | 212 |
| Missing Value Handling | Definition of Missing | User defined missing values are treated as missing. |
| Cases Used | Statistics for each analysis are based on the cases with no missing or out-of-range data for any variable in the analysis. |
| Syntax | | T-TEST  GROUPS = sleep\_4cat(1 2)  /MISSING = ANALYSIS  /VARIABLES = a\_hads\_a  /CRITERIA = CI(.95) . |
| Resources | Elapsed Time | 0:00:00,04 |

  


Group Statistics

|  | sleep\_4cat | N | Mean | Std. Deviation | Std. Error Mean |
| a\_hads\_a | 1,00 | 131 | 5,12 | 3,148 | ,275 |
| 2,00 | 22 | 6,77 | 4,898 | 1,044 |

  


Independent Samples Test

|  |  | Levene's Test for Equality of Variances | | t-test for Equality of Means | | | | | | |
| F | Sig. | t | df | Sig. (2-tailed) | Mean Difference | Std. Error Difference | 95% Confidence Interval of the Difference | |
| Lower | Upper |
| a\_hads\_a | Equal variances assumed | 6,758 | ,010 | -2,080 | 151 | ,039 | -1,651 | ,794 | -3,219 | -,082 |
| Equal variances not assumed |  |  | -1,528 | 23,995 | ,139 | -1,651 | 1,080 | -3,879 | ,578 |

  


# T-Test


Notes

| Output Created | | 22-JAN-2021 18:56:23 |
| Comments | |  |
| Input | Data | C:\Users\Barbara\cro\analisi\_dati\survivors\_linfomi\_dati2020\database\_12\_gennaio\_2021\dati\_12\_gennaio\_2021.sav |
| Filter | <none> |
| Weight | <none> |
| Split File | <none> |
| N of Rows in Working Data File | 212 |
| Missing Value Handling | Definition of Missing | User defined missing values are treated as missing. |
| Cases Used | Statistics for each analysis are based on the cases with no missing or out-of-range data for any variable in the analysis. |
| Syntax | | T-TEST  GROUPS = sleep\_4cat(1 3)  /MISSING = ANALYSIS  /VARIABLES = a\_hads\_a  /CRITERIA = CI(.95) . |
| Resources | Elapsed Time | 0:00:00,07 |

  


Group Statistics

|  | sleep\_4cat | N | Mean | Std. Deviation | Std. Error Mean |
| a\_hads\_a | 1,00 | 131 | 5,12 | 3,148 | ,275 |
| 3,00 | 47 | 6,21 | 4,070 | ,594 |

  


Independent Samples Test

|  |  | Levene's Test for Equality of Variances | | t-test for Equality of Means | | | | | | |
| F | Sig. | t | df | Sig. (2-tailed) | Mean Difference | Std. Error Difference | 95% Confidence Interval of the Difference | |
| Lower | Upper |
| a\_hads\_a | Equal variances assumed | 6,079 | ,015 | -1,879 | 176 | ,062 | -1,091 | ,580 | -2,236 | ,055 |
| Equal variances not assumed |  |  | -1,667 | 66,777 | ,100 | -1,091 | ,654 | -2,397 | ,215 |

  


# T-Test


Notes

| Output Created | | 22-JAN-2021 18:56:23 |
| Comments | |  |
| Input | Data | C:\Users\Barbara\cro\analisi\_dati\survivors\_linfomi\_dati2020\database\_12\_gennaio\_2021\dati\_12\_gennaio\_2021.sav |
| Filter | <none> |
| Weight | <none> |
| Split File | <none> |
| N of Rows in Working Data File | 212 |
| Missing Value Handling | Definition of Missing | User defined missing values are treated as missing. |
| Cases Used | Statistics for each analysis are based on the cases with no missing or out-of-range data for any variable in the analysis. |
| Syntax | | T-TEST  GROUPS = sleep\_4cat(1 4)  /MISSING = ANALYSIS  /VARIABLES = a\_hads\_a  /CRITERIA = CI(.95) . |
| Resources | Elapsed Time | 0:00:00,04 |

  


Group Statistics

|  | sleep\_4cat | N | Mean | Std. Deviation | Std. Error Mean |
| a\_hads\_a | 1,00 | 131 | 5,12 | 3,148 | ,275 |
| 4,00 | 12 | 8,42 | 4,166 | 1,203 |

  


Independent Samples Test

|  |  | Levene's Test for Equality of Variances | | t-test for Equality of Means | | | | | | |
| F | Sig. | t | df | Sig. (2-tailed) | Mean Difference | Std. Error Difference | 95% Confidence Interval of the Difference | |
| Lower | Upper |
| a\_hads\_a | Equal variances assumed | 2,525 | ,114 | -3,373 | 141 | ,001 | -3,295 | ,977 | -5,226 | -1,363 |
| Equal variances not assumed |  |  | -2,670 | 12,178 | ,020 | -3,295 | 1,234 | -5,978 | -,611 |

  


# T-Test


Notes

| Output Created | | 22-JAN-2021 18:56:23 |
| Comments | |  |
| Input | Data | C:\Users\Barbara\cro\analisi\_dati\survivors\_linfomi\_dati2020\database\_12\_gennaio\_2021\dati\_12\_gennaio\_2021.sav |
| Filter | <none> |
| Weight | <none> |
| Split File | <none> |
| N of Rows in Working Data File | 212 |
| Missing Value Handling | Definition of Missing | User defined missing values are treated as missing. |
| Cases Used | Statistics for each analysis are based on the cases with no missing or out-of-range data for any variable in the analysis. |
| Syntax | | T-TEST  GROUPS = sleep\_4cat(2 3)  /MISSING = ANALYSIS  /VARIABLES = a\_hads\_a  /CRITERIA = CI(.95) . |
| Resources | Elapsed Time | 0:00:00,04 |

  


Group Statistics

|  | sleep\_4cat | N | Mean | Std. Deviation | Std. Error Mean |
| a\_hads\_a | 2,00 | 22 | 6,77 | 4,898 | 1,044 |
| 3,00 | 47 | 6,21 | 4,070 | ,594 |

  


Independent Samples Test

|  |  | Levene's Test for Equality of Variances | | t-test for Equality of Means | | | | | | |
| F | Sig. | t | df | Sig. (2-tailed) | Mean Difference | Std. Error Difference | 95% Confidence Interval of the Difference | |
| Lower | Upper |
| a\_hads\_a | Equal variances assumed | ,402 | ,528 | ,499 | 67 | ,620 | ,560 | 1,123 | -1,681 | 2,801 |
| Equal variances not assumed |  |  | ,466 | 35,091 | ,644 | ,560 | 1,201 | -1,878 | 2,998 |

  


# T-Test


Notes

| Output Created | | 22-JAN-2021 18:56:23 |
| Comments | |  |
| Input | Data | C:\Users\Barbara\cro\analisi\_dati\survivors\_linfomi\_dati2020\database\_12\_gennaio\_2021\dati\_12\_gennaio\_2021.sav |
| Filter | <none> |
| Weight | <none> |
| Split File | <none> |
| N of Rows in Working Data File | 212 |
| Missing Value Handling | Definition of Missing | User defined missing values are treated as missing. |
| Cases Used | Statistics for each analysis are based on the cases with no missing or out-of-range data for any variable in the analysis. |
| Syntax | | T-TEST  GROUPS = sleep\_4cat(2 4)  /MISSING = ANALYSIS  /VARIABLES = a\_hads\_a  /CRITERIA = CI(.95) . |
| Resources | Elapsed Time | 0:00:00,03 |

  


Group Statistics

|  | sleep\_4cat | N | Mean | Std. Deviation | Std. Error Mean |
| a\_hads\_a | 2,00 | 22 | 6,77 | 4,898 | 1,044 |
| 4,00 | 12 | 8,42 | 4,166 | 1,203 |

  


Independent Samples Test

|  |  | Levene's Test for Equality of Variances | | t-test for Equality of Means | | | | | | |
| F | Sig. | t | df | Sig. (2-tailed) | Mean Difference | Std. Error Difference | 95% Confidence Interval of the Difference | |
| Lower | Upper |
| a\_hads\_a | Equal variances assumed | ,114 | ,738 | -,983 | 32 | ,333 | -1,644 | 1,672 | -5,050 | 1,762 |
| Equal variances not assumed |  |  | -1,032 | 26,077 | ,311 | -1,644 | 1,593 | -4,917 | 1,630 |

  


# T-Test


Notes

| Output Created | | 22-JAN-2021 18:56:23 |
| Comments | |  |
| Input | Data | C:\Users\Barbara\cro\analisi\_dati\survivors\_linfomi\_dati2020\database\_12\_gennaio\_2021\dati\_12\_gennaio\_2021.sav |
| Filter | <none> |
| Weight | <none> |
| Split File | <none> |
| N of Rows in Working Data File | 212 |
| Missing Value Handling | Definition of Missing | User defined missing values are treated as missing. |
| Cases Used | Statistics for each analysis are based on the cases with no missing or out-of-range data for any variable in the analysis. |
| Syntax | | T-TEST  GROUPS = sleep\_4cat(3 4)  /MISSING = ANALYSIS  /VARIABLES = a\_hads\_a  /CRITERIA = CI(.95) . |
| Resources | Elapsed Time | 0:00:00,03 |

  


Group Statistics

|  | sleep\_4cat | N | Mean | Std. Deviation | Std. Error Mean |
| a\_hads\_a | 3,00 | 47 | 6,21 | 4,070 | ,594 |
| 4,00 | 12 | 8,42 | 4,166 | 1,203 |

  


Independent Samples Test

|  |  | Levene's Test for Equality of Variances | | t-test for Equality of Means | | | | | | |
| F | Sig. | t | df | Sig. (2-tailed) | Mean Difference | Std. Error Difference | 95% Confidence Interval of the Difference | |
| Lower | Upper |
| a\_hads\_a | Equal variances assumed | ,012 | ,912 | -1,667 | 57 | ,101 | -2,204 | 1,322 | -4,852 | ,444 |
| Equal variances not assumed |  |  | -1,643 | 16,775 | ,119 | -2,204 | 1,341 | -5,036 | ,629 |

  
