# Supplementary material for: Cancer-Related Psychological Distress in Lymphoma Survivor: An Italian Cross-Sectional Study
Source: Front Psychol. 2022 Apr 26;13:872329. doi: 10.3389/fpsyg.2022.872329 (PMC9088809; doi:10.3389/fpsyg.2022.872329)
Supplement: Supplementary file 1 [file Data_Sheet_1.zip › STATISTIC ANALYSIS/27B_POST_HOC__T-Test_SLEEP-D.HTM]

<!--Text used as the document title (displayed in the title bar).-->


# T-Test


Notes

| Output Created | | 22-JAN-2021 19:03:00 |
| Comments | |  |
| Input | Data | C:\Users\Barbara\cro\analisi\_dati\survivors\_linfomi\_dati2020\database\_12\_gennaio\_2021\dati\_12\_gennaio\_2021.sav |
| Filter | <none> |
| Weight | <none> |
| Split File | <none> |
| N of Rows in Working Data File | 212 |
| Missing Value Handling | Definition of Missing | User defined missing values are treated as missing. |
| Cases Used | Statistics for each analysis are based on the cases with no missing or out-of-range data for any variable in the analysis. |
| Syntax | | T-TEST  GROUPS = sleep\_4cat(1 2)  /MISSING = ANALYSIS  /VARIABLES = a\_hads\_d  /CRITERIA = CI(.95) . |
| Resources | Elapsed Time | 0:00:00,04 |

  


Group Statistics

|  | sleep\_4cat | N | Mean | Std. Deviation | Std. Error Mean |
| a\_hads\_d | 1,00 | 131 | 3,34 | 2,523 | ,220 |
| 2,00 | 22 | 5,59 | 3,261 | ,695 |

  


Independent Samples Test

|  |  | Levene's Test for Equality of Variances | | t-test for Equality of Means | | | | | | |
| F | Sig. | t | df | Sig. (2-tailed) | Mean Difference | Std. Error Difference | 95% Confidence Interval of the Difference | |
| Lower | Upper |
| a\_hads\_d | Equal variances assumed | 5,496 | ,020 | -3,711 | 151 | ,000 | -2,255 | ,608 | -3,456 | -1,054 |
| Equal variances not assumed |  |  | -3,092 | 25,391 | ,005 | -2,255 | ,729 | -3,756 | -,754 |

  


# T-Test


Notes

| Output Created | | 22-JAN-2021 19:03:00 |
| Comments | |  |
| Input | Data | C:\Users\Barbara\cro\analisi\_dati\survivors\_linfomi\_dati2020\database\_12\_gennaio\_2021\dati\_12\_gennaio\_2021.sav |
| Filter | <none> |
| Weight | <none> |
| Split File | <none> |
| N of Rows in Working Data File | 212 |
| Missing Value Handling | Definition of Missing | User defined missing values are treated as missing. |
| Cases Used | Statistics for each analysis are based on the cases with no missing or out-of-range data for any variable in the analysis. |
| Syntax | | T-TEST  GROUPS = sleep\_4cat(1 3)  /MISSING = ANALYSIS  /VARIABLES = a\_hads\_d  /CRITERIA = CI(.95) . |
| Resources | Elapsed Time | 0:00:00,03 |

  


Group Statistics

|  | sleep\_4cat | N | Mean | Std. Deviation | Std. Error Mean |
| a\_hads\_d | 1,00 | 131 | 3,34 | 2,523 | ,220 |
| 3,00 | 47 | 4,77 | 3,583 | ,523 |

  


Independent Samples Test

|  |  | Levene's Test for Equality of Variances | | t-test for Equality of Means | | | | | | |
| F | Sig. | t | df | Sig. (2-tailed) | Mean Difference | Std. Error Difference | 95% Confidence Interval of the Difference | |
| Lower | Upper |
| a\_hads\_d | Equal variances assumed | 5,150 | ,024 | -2,964 | 176 | ,003 | -1,430 | ,483 | -2,382 | -,478 |
| Equal variances not assumed |  |  | -2,521 | 63,113 | ,014 | -1,430 | ,567 | -2,563 | -,297 |

  


# T-Test


Notes

| Output Created | | 22-JAN-2021 19:03:00 |
| Comments | |  |
| Input | Data | C:\Users\Barbara\cro\analisi\_dati\survivors\_linfomi\_dati2020\database\_12\_gennaio\_2021\dati\_12\_gennaio\_2021.sav |
| Filter | <none> |
| Weight | <none> |
| Split File | <none> |
| N of Rows in Working Data File | 212 |
| Missing Value Handling | Definition of Missing | User defined missing values are treated as missing. |
| Cases Used | Statistics for each analysis are based on the cases with no missing or out-of-range data for any variable in the analysis. |
| Syntax | | T-TEST  GROUPS = sleep\_4cat(1 4)  /MISSING = ANALYSIS  /VARIABLES = a\_hads\_d  /CRITERIA = CI(.95) . |
| Resources | Elapsed Time | 0:00:00,04 |

  


Group Statistics

|  | sleep\_4cat | N | Mean | Std. Deviation | Std. Error Mean |
| a\_hads\_d | 1,00 | 131 | 3,34 | 2,523 | ,220 |
| 4,00 | 12 | 5,58 | 2,429 | ,701 |

  


Independent Samples Test

|  |  | Levene's Test for Equality of Variances | | t-test for Equality of Means | | | | | | |
| F | Sig. | t | df | Sig. (2-tailed) | Mean Difference | Std. Error Difference | 95% Confidence Interval of the Difference | |
| Lower | Upper |
| a\_hads\_d | Equal variances assumed | ,316 | ,575 | -2,962 | 141 | ,004 | -2,247 | ,759 | -3,747 | -,748 |
| Equal variances not assumed |  |  | -3,057 | 13,269 | ,009 | -2,247 | ,735 | -3,832 | -,663 |

  


# T-Test


Notes

| Output Created | | 22-JAN-2021 19:03:00 |
| Comments | |  |
| Input | Data | C:\Users\Barbara\cro\analisi\_dati\survivors\_linfomi\_dati2020\database\_12\_gennaio\_2021\dati\_12\_gennaio\_2021.sav |
| Filter | <none> |
| Weight | <none> |
| Split File | <none> |
| N of Rows in Working Data File | 212 |
| Missing Value Handling | Definition of Missing | User defined missing values are treated as missing. |
| Cases Used | Statistics for each analysis are based on the cases with no missing or out-of-range data for any variable in the analysis. |
| Syntax | | T-TEST  GROUPS = sleep\_4cat(2 3)  /MISSING = ANALYSIS  /VARIABLES = a\_hads\_d  /CRITERIA = CI(.95) . |
| Resources | Elapsed Time | 0:00:00,06 |

  


Group Statistics

|  | sleep\_4cat | N | Mean | Std. Deviation | Std. Error Mean |
| a\_hads\_d | 2,00 | 22 | 5,59 | 3,261 | ,695 |
| 3,00 | 47 | 4,77 | 3,583 | ,523 |

  


Independent Samples Test

|  |  | Levene's Test for Equality of Variances | | t-test for Equality of Means | | | | | | |
| F | Sig. | t | df | Sig. (2-tailed) | Mean Difference | Std. Error Difference | 95% Confidence Interval of the Difference | |
| Lower | Upper |
| a\_hads\_d | Equal variances assumed | ,063 | ,803 | ,916 | 67 | ,363 | ,825 | ,900 | -,972 | 2,622 |
| Equal variances not assumed |  |  | ,948 | 44,891 | ,348 | ,825 | ,870 | -,927 | 2,577 |

  


# T-Test


Notes

| Output Created | | 22-JAN-2021 19:03:00 |
| Comments | |  |
| Input | Data | C:\Users\Barbara\cro\analisi\_dati\survivors\_linfomi\_dati2020\database\_12\_gennaio\_2021\dati\_12\_gennaio\_2021.sav |
| Filter | <none> |
| Weight | <none> |
| Split File | <none> |
| N of Rows in Working Data File | 212 |
| Missing Value Handling | Definition of Missing | User defined missing values are treated as missing. |
| Cases Used | Statistics for each analysis are based on the cases with no missing or out-of-range data for any variable in the analysis. |
| Syntax | | T-TEST  GROUPS = sleep\_4cat(2 4)  /MISSING = ANALYSIS  /VARIABLES = a\_hads\_d  /CRITERIA = CI(.95) . |
| Resources | Elapsed Time | 0:00:00,04 |

  


Group Statistics

|  | sleep\_4cat | N | Mean | Std. Deviation | Std. Error Mean |
| a\_hads\_d | 2,00 | 22 | 5,59 | 3,261 | ,695 |
| 4,00 | 12 | 5,58 | 2,429 | ,701 |

  


Independent Samples Test

|  |  | Levene's Test for Equality of Variances | | t-test for Equality of Means | | | | | | |
| F | Sig. | t | df | Sig. (2-tailed) | Mean Difference | Std. Error Difference | 95% Confidence Interval of the Difference | |
| Lower | Upper |
| a\_hads\_d | Equal variances assumed | 3,802 | ,060 | ,007 | 32 | ,994 | ,008 | 1,077 | -2,186 | 2,201 |
| Equal variances not assumed |  |  | ,008 | 28,718 | ,994 | ,008 | ,988 | -2,013 | 2,028 |

  


# T-Test


Notes

| Output Created | | 22-JAN-2021 19:03:00 |
| Comments | |  |
| Input | Data | C:\Users\Barbara\cro\analisi\_dati\survivors\_linfomi\_dati2020\database\_12\_gennaio\_2021\dati\_12\_gennaio\_2021.sav |
| Filter | <none> |
| Weight | <none> |
| Split File | <none> |
| N of Rows in Working Data File | 212 |
| Missing Value Handling | Definition of Missing | User defined missing values are treated as missing. |
| Cases Used | Statistics for each analysis are based on the cases with no missing or out-of-range data for any variable in the analysis. |
| Syntax | | T-TEST  GROUPS = sleep\_4cat(3 4)  /MISSING = ANALYSIS  /VARIABLES = a\_hads\_d  /CRITERIA = CI(.95) . |
| Resources | Elapsed Time | 0:00:00,03 |

  


Group Statistics

|  | sleep\_4cat | N | Mean | Std. Deviation | Std. Error Mean |
| a\_hads\_d | 3,00 | 47 | 4,77 | 3,583 | ,523 |
| 4,00 | 12 | 5,58 | 2,429 | ,701 |

  


Independent Samples Test

|  |  | Levene's Test for Equality of Variances | | t-test for Equality of Means | | | | | | |
| F | Sig. | t | df | Sig. (2-tailed) | Mean Difference | Std. Error Difference | 95% Confidence Interval of the Difference | |
| Lower | Upper |
| a\_hads\_d | Equal variances assumed | 1,728 | ,194 | -,745 | 57 | ,459 | -,817 | 1,097 | -3,013 | 1,379 |
| Equal variances not assumed |  |  | -,935 | 24,781 | ,359 | -,817 | ,875 | -2,619 | ,985 |

  
