# Supplementary material for: Cancer-Related Psychological Distress in Lymphoma Survivor: An Italian Cross-Sectional Study
Source: Front Psychol. 2022 Apr 26;13:872329. doi: 10.3389/fpsyg.2022.872329 (PMC9088809; doi:10.3389/fpsyg.2022.872329)
Supplement: Supplementary file 1 [file Data_Sheet_1.zip › STATISTIC ANALYSIS/27_Oneway_SLEEP-A_D.HTM]

<!--Text used as the document title (displayed in the title bar).-->


# Oneway


Notes

| Output Created | | 22-JAN-2021 18:47:46 |
| Comments | |  |
| Input | Data | C:\Users\Barbara\cro\analisi\_dati\survivors\_linfomi\_dati2020\database\_12\_gennaio\_2021\dati\_12\_gennaio\_2021.sav |
| Filter | <none> |
| Weight | <none> |
| Split File | <none> |
| N of Rows in Working Data File | 212 |
| Missing Value Handling | Definition of Missing | User-defined missing values are treated as missing. |
| Cases Used | Statistics for each analysis are based on cases with no missing data for any variable in the analysis. |
| Syntax | | ONEWAY  a\_hads\_a a\_hads\_d BY sleep\_4cat  /STATISTICS DESCRIPTIVES  /MISSING ANALYSIS . |
| Resources | Elapsed Time | 0:00:00,05 |

  


Descriptives

|  |  | N | Mean | Std. Deviation | Std. Error | 95% Confidence Interval for Mean | | Minimum | Maximum |
| Lower Bound | Upper Bound |  
  

| a\_hads\_a | 1,00 | 131 | 5,12 | 3,148 | ,275 | 4,58 | 5,67 | 0 | 16 |
| 2,00 | 22 | 6,77 | 4,898 | 1,044 | 4,60 | 8,94 | 0 | 18 |
| 3,00 | 47 | 6,21 | 4,070 | ,594 | 5,02 | 7,41 | 0 | 14 |
| 4,00 | 12 | 8,42 | 4,166 | 1,203 | 5,77 | 11,06 | 0 | 14 |
| Total | 212 | 5,72 | 3,717 | ,255 | 5,22 | 6,22 | 0 | 18 |
| a\_hads\_d | 1,00 | 131 | 3,34 | 2,523 | ,220 | 2,90 | 3,77 | 0 | 13 |
| 2,00 | 22 | 5,59 | 3,261 | ,695 | 4,15 | 7,04 | 2 | 11 |
| 3,00 | 47 | 4,77 | 3,583 | ,523 | 3,71 | 5,82 | 0 | 16 |
| 4,00 | 12 | 5,58 | 2,429 | ,701 | 4,04 | 7,13 | 2 | 11 |
| Total | 212 | 4,01 | 2,983 | ,205 | 3,61 | 4,42 | 0 | 16 |

  


ANOVA

|  |  | Sum of Squares | df | Mean Square | F | Sig. |
| a\_hads\_a | Between Groups | 169,882 | 3 | 56,627 | 4,291 | ,006 |
| Within Groups | 2744,698 | 208 | 13,196 |  |  |
| Total | 2914,580 | 211 |  |  |  |
| a\_hads\_d | Between Groups | 171,076 | 3 | 57,025 | 6,953 | ,000 |
| Within Groups | 1705,882 | 208 | 8,201 |  |  |
| Total | 1876,958 | 211 |  |  |  |

  
