# Supplementary material for: Cancer-Related Psychological Distress in Lymphoma Survivor: An Italian Cross-Sectional Study
Source: Front Psychol. 2022 Apr 26;13:872329. doi: 10.3389/fpsyg.2022.872329 (PMC9088809; doi:10.3389/fpsyg.2022.872329)
Supplement: Supplementary file 1 [file Data_Sheet_1.zip › STATISTIC ANALYSIS/28_T-Test_PSYCHIATRIC DRUGS_A-D.HTM]

<!--Text used as the document title (displayed in the title bar).-->


# T-Test


Notes

| Output Created | | 22-JAN-2021 19:23:26 |
| Comments | |  |
| Input | Data | C:\Users\Barbara\cro\analisi\_dati\survivors\_linfomi\_dati2020\database\_12\_gennaio\_2021\dati\_12\_gennaio\_2021.sav |
| Filter | <none> |
| Weight | <none> |
| Split File | <none> |
| N of Rows in Working Data File | 212 |
| Missing Value Handling | Definition of Missing | User defined missing values are treated as missing. |
| Cases Used | Statistics for each analysis are based on the cases with no missing or out-of-range data for any variable in the analysis. |
| Syntax | | T-TEST  GROUPS = psicofarmaci\_dic(1 2)  /MISSING = ANALYSIS  /VARIABLES = a\_hads\_a a\_hads\_d  /CRITERIA = CI(.95) . |
| Resources | Elapsed Time | 0:00:00,05 |

  


Group Statistics

|  | psicofarmaci\_dic | N | Mean | Std. Deviation | Std. Error Mean |
| a\_hads\_a | 1,00 | 169 | 5,07 | 3,257 | ,251 |
| 2,00 | 43 | 8,30 | 4,290 | ,654 |
| a\_hads\_d | 1,00 | 169 | 3,50 | 2,695 | ,207 |
| 2,00 | 43 | 6,02 | 3,233 | ,493 |

  


Independent Samples Test

|  |  | Levene's Test for Equality of Variances | | t-test for Equality of Means | | | | | | |
| F | Sig. | t | df | Sig. (2-tailed) | Mean Difference | Std. Error Difference | 95% Confidence Interval of the Difference | |
| Lower | Upper |
| a\_hads\_a | Equal variances assumed | 6,710 | ,010 | -5,433 | 210 | ,000 | -3,237 | ,596 | -4,412 | -2,063 |
| Equal variances not assumed |  |  | -4,621 | 54,926 | ,000 | -3,237 | ,701 | -4,641 | -1,833 |
| a\_hads\_d | Equal variances assumed | 3,460 | ,064 | -5,250 | 210 | ,000 | -2,520 | ,480 | -3,467 | -1,574 |
| Equal variances not assumed |  |  | -4,712 | 57,715 | ,000 | -2,520 | ,535 | -3,591 | -1,450 |

  
