# Supplementary material for: Cancer-Related Psychological Distress in Lymphoma Survivor: An Italian Cross-Sectional Study
Source: Front Psychol. 2022 Apr 26;13:872329. doi: 10.3389/fpsyg.2022.872329 (PMC9088809; doi:10.3389/fpsyg.2022.872329)
Supplement: Supplementary file 1 [file Data_Sheet_1.zip › STATISTIC ANALYSIS/30_T_PAIRED_ANSIA_DWEPRESSIONE.HTM]

<!--Text used as the document title (displayed in the title bar).-->


# T-Test


Notes

| Output Created | | 22-MAR-2021 19:20:54 |
| Comments | |  |
| Input | Data | C:\Users\Barbara\cro\analisi\_dati\survivors\_linfomi\_dati2020\database\_12\_gennaio\_2021\dati\_12\_gennaio\_2021.sav |
| Filter | <none> |
| Weight | <none> |
| Split File | <none> |
| N of Rows in Working Data File | 212 |
| Missing Value Handling | Definition of Missing | User defined missing values are treated as missing. |
| Cases Used | Statistics for each analysis are based on the cases with no missing or out-of-range data for any variable in the analysis. |
| Syntax | | T-TEST  PAIRS = a\_hads\_a WITH a\_hads\_d (PAIRED)  /CRITERIA = CI(.95)  /MISSING = ANALYSIS. |
| Resources | Elapsed Time | 0:00:00,05 |

  


Paired Samples Statistics

|  |  | Mean | N | Std. Deviation | Std. Error Mean |
| Pair 1 | a\_hads\_a | 5,72 | 212 | 3,717 | ,255 |
| a\_hads\_d | 4,01 | 212 | 2,983 | ,205 |

  


Paired Samples Correlations

|  |  | N | Correlation | Sig. |
| Pair 1 | a\_hads\_a & a\_hads\_d | 212 | ,711 | ,000 |

  


Paired Samples Test

|  |  | Paired Differences | | | | | t | df | Sig. (2-tailed) |
| Mean | Std. Deviation | Std. Error Mean | 95% Confidence Interval of the Difference | |  
  
  

| Lower | Upper |  
  
  

| Pair 1 | a\_hads\_a - a\_hads\_d | 1,708 | 2,634 | ,181 | 1,351 | 2,064 | 9,439 | 211 | ,000 |

  
